# Design and Synthesis of Potent *N*-Acylethanolamine-hydrolyzing Acid Amidase (NAAA) Inhibitor as Anti-Inflammatory Compounds

**DOI:** 10.1371/journal.pone.0043023

**Published:** 2012-08-20

**Authors:** Yuhang Li, Longhe Yang, Ling Chen, Chenggang Zhu, Rui Huang, Xiao Zheng, Yan Qiu, Jin Fu

**Affiliations:** 1 Department of Medical Sciences, Medical College, Xiamen University, Xiamen, Fujian, China; 2 Department of Chemistry and The Key Laboratory for Chemical Biology of Fujian Province, College of Chemistry and Chemical Engineering, Xiamen University, Xiamen, Fujian, China; 3 Drug Discovery and Development, Italian Institute of Technology, Genoa, Italy; Medical School of Hannover, United States of America

## Abstract

*N*-acylethanolamine-hydrolyzing acid amidase (NAAA) is a lysosomal enzyme involved in biological deactivation of *N*-palmitoylethanolamide (PEA), which exerts anti-inflammatory and analgesic effects through the activation of nuclear receptor peroxisome proliferator-activated receptor-alpha (PPAR-α). To develop selective and potent NAAA inhibitors, we designed and synthesized a series of derivatives of 1-pentadecanyl-carbonyl pyrrolidine (compound **1**), a general amidase inhibitor. Structure activity relationship (SAR) studies have identified a compound **16**, 1-(2-Biphenyl-4-yl)ethyl-carbonyl pyrrolidine, which has shown the highest inhibition on NAAA activity (IC_50_ = 2.12±0.41 µM) and is characterized as a reversible and competitive NAAA inhibitor. Computational docking analysis and mutagenesis study revealed that compound **16** interacted with Asparagine 209 (Asn^209^) residue flanking the catalytic pocket of NAAA so as to block the substrate entrance. *In vitro* pharmacological studies demonstrated that compound **16** dose-dependently reduced mRNA expression levels of iNOS and IL-6, along with an increase of intracellular PEA levels, in mouse macrophages with lipopolysaccharides (LPS) induced inflammation. Our study discovered a novel NAAA inhibitor, compound **16,** that could serve as a potential anti-inflammatory agent.

## Introduction

**Figure 1:**
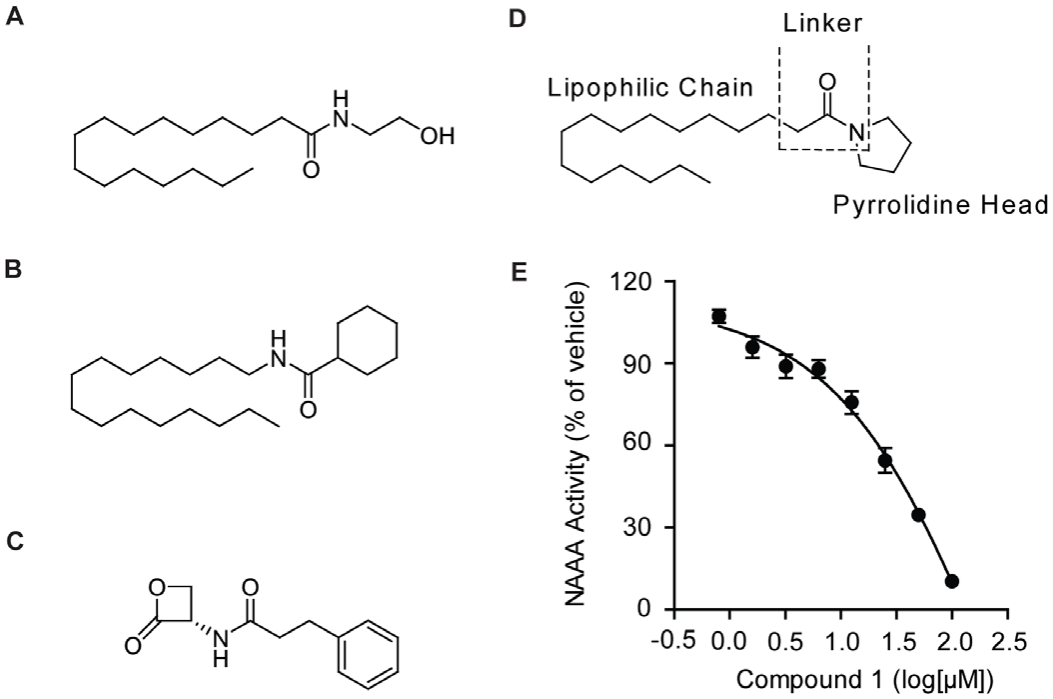
The strategy of developing NAAA inhibitors. A–C, the chemical structures of classic NAAA inhibitors including PEA (A), CCP (B), and (S)-OOPP (C); (D) SAR study of 1-Pentadecanyl-carbonyl pyrrolidine; (E) Dose-dependent inhibition of 1-Pentadecanyl-carbonyl pyrrolidine on NAAA activity.

Palmitoylethanolamide (PEA) (Figure 1A) is an endogenous fatty acid ethanolamide (FAE) expressed in many mammalian tissues. It has demonstrated anti-inflammatory [1], [2], [3] and analgesia [4], [5] effects through the activation of nuclear receptor peroxisome proliferator-activated receptor-alpha (PPAR-α) [6]. The endogenous levels of PEA in animal tissues are controlled by enzymes responsible for its formation and degradation. PEA is synthesized from a phospholipid precursor of *N*-palmitoylphosphatidylethanolamine (NAPE) catalyzed by NAPE-specific phospholipase D [7] and degraded to palmitic acid by the deactivation enzymes, i.e., *N*-acylethanolamine-hydrolyzing acid amidase (NAAA) [8] and fatty acid amide hydrolase (FAAH) [9].

FAAH is a membrane-bound protein responsible for fatty acid ethanolamide (FAE) hydrolysis [9]. FAAH inhibitors have been extensively studied and they have exhibited broad pharmacological properties [10], [11], [12]. In contrast to FAAH, NAAA is a subcellular protein located in lysosome and its bioactivity is optimal at pH of 4.5–5.0 [8]. Though both FAAH and NAAA can hydrolyze various FAEs, their molecular homologues show no similarity and the substrate preferences are quite different as well [8]. NAAA shows high reactivity to PEA, while FAAH prefers anandamide. NAAA is a N-terminal nucleophile (Ntn) hydrolase that catalyzes the degradation of several non-peptide C-N bonds [13]. The N-terminal self cleavage is a critical action during the enzyme activation [14], and cysteine 131 (cys^131^) is suggested to be nucleophile residue that forms the catalytic pocket with other amino acids, such as Aspartic acid (Asp^150^), Tyrosine (Tyr^151^) and Asparagine (Asn^292^) [15]. NAAA is wildly expressed in many tissues, especially those associated with immune responses, e.g., lung, spleen and small intestine [15], and exhibits significant anti-inflammatory properties [3], [16].

Although the biochemical characteristics of NAAA have been intensively studied, there are only two classes of NAAA inhibitors thus far identified. One class of NAAA inhibitors are PEA derivatives [17], [18], such as *N*-Cyclohexanecarbonylpentadecylamine (CCP) (Figure 1B), which serves as a reversible and non-competitive NAAA inhibitor. Another class of NAAA inhibitors are β-lactone compounds, such as (*S*)-*N*-(2-oxo-3-oxetanyl)-3-phenylpropionamide ((*S*)-OOPP) (Figure 1C), which is about 10-fold more potent than CCP [3]. Though (*S*)-OOPP has demonstrated profound anti-inflammatory effect in several animal models [3], the labile β-lactone structure limits its drug potential. Therefore, it is desirable to develop and discover novel NAAA inhibitors with stable backbone structures. In present study, we designed and identified a novel potent NAAA inhibitor, a derivative of 1-pentadecanyl-carbonyl pyrrolidine (Figure 1D, compound **1**), via SAR screening. Furthermore, we characterized the pharmacological effects of this compound and investigated its anti-inflammatory properties.

## Results

### Structure Activity Relationship (SAR) Studies for Pyrrolidine Derivatives

Based on the NAAA catalytic activation site, we designed and synthesized a series of PEA derivatives (Table S1) and performed the enzymatic assays in terms of LC/MS methodology. The data showed that 1-pentadecanyl-carbonyl pyrrolidine (compound **1**) exhibited inhibition on NAAA activity with IC_50_ = 25.01±5.70 µM (Figure 1E & Table S1), and on FAAH activity with IC_50_ = 21.78±4.45 µM (Table S1). Compound **1** was thence used as the backbone for hit-to-lead optimization in our study to develop potent and selective NAAA inhibitors.

According to the chemical structure of compound **1** (Figure 1D), there are three regions, i.e., the lipophilic chain, the pyrrolidine head, and the linker, which may be modified pursuant to our objectives. SAR study on compound **1** focused on its pyrrolidine head ring and acyl chain. First, we replaced the pyrrolidine head with cyclopentanamine (compound **2**), piperidine (compound **3**), cyclopentanol (compound **4**), tetrahydrofuran-3-ol (compound **5**) or pyrrole (compound **6**), all of which resulted in a complete loss of the inhibition ability on NAAA activity and FAAH activity (Table S1). Next, we introduced lipophilic group to modify fatty acid chain, e.g., replacing alkyl chain with benzyl to increase rigidity. Table S2 listed the lipophilic chain Ph(CH_2_)_n_ used to substitute the saturated alkyl chain n-C_14_H_29_ of compound **1** (Table S2, compound **7**–**13**). Enzymatic assay indicated an increase of inhibition on NAAA activity when compounds contained longer carbon chains (n ≥4) and the most potent effect was achieved with IC_50_ = 12.92±3.47 µM when carbon chain assuming n = 6 (compound **12**). Compound **12** bearing Ph(CH_2_)_6_− group was subsequently subject to further SAR study and optimization. Three series of aryl-containing lipophilic chains were therefore introduced and examined (Table S2, compound **14**–**20**), and compound **16**, 1-(2-Biphenyl-4-yl)ethyl-carbonyl pyrrolidine, exhibited a potent inhibition on NAAA activity with IC_50_ = 2.12±0.41 µM. In addition, comparing to its inhibition on NAAA activity, compound **16** demonstrated much lower inhibitory effect on FAAH activity, i.e., reducing no more than 30% of FAAH activity even at concentration of 100 µM, and had no inhibitory effect on other lipid-hydrolyzing enzymes, e.g., monoacylglycerol lipase (MGL) and *N*-acylsphingosine amidohydrolase (ASAH) (Figure S1).

Based on compound **16**, we further pursued SAR study on the linker between pyrrolidine and lipophilic groups (Table S3). Substitutions of its amide group by urea (compound **21**), amino carbamate (compound **22**), retroamide (compound **23**), ester (compound **24**), or amine (compound **25**) resulted in a full loss of inhibition on NAAA and FAAH activities (Table S3).

### Interaction of Compound 16 with NAAA

**Figure 2:**
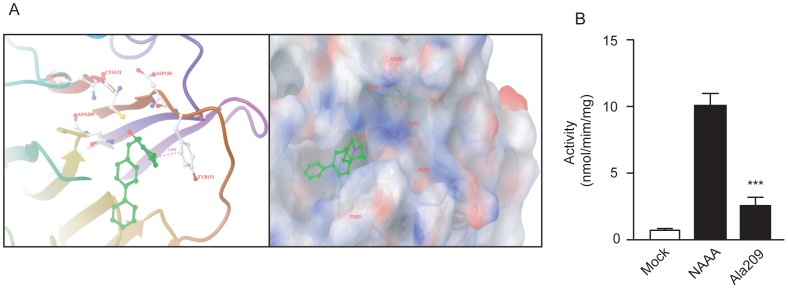
Compound 16 interacted with NAAA protein. (A) Computational model illustrated docking of compound 16 at the active site of rat NAAA. (B) Effect of mutant Ala^209^-NAAA on NAAA activity. Mock, HEK293 cell heterogeneously overexpressing vector control; NAAA, HEK293 cell heterogeneously overexpressing NAAA; Ala209, HEK293 cell heterogeneously overexpressing mutant Ala^209^-NAAA. ***, P<0.001 vs. NAAA, n = 5.

Solorzano *et al*. [16] have previously described a 3-dimensional model of NAAA catalytic site based on the crystal structure of another Ntn cysteine hydrolase-conjugated bile acid hydrolase (CBAH), which shares a highly conserved sequence with NAAA protein in the catalytic N-terminal region. To test whether compound **16** interacted with the activation site of NAAA, we utilized this computational model to characterize the binding pocket of compound **16**. Figure 2A showed that compound **16** posed into the NAAA catalytic pocket and formed a hydrogen bond with Asn^209^, suggesting that Asn^209^ be a critical residue engaged in NAAA activity. To verify this predicted model, we mutated Asn^209^ residue to Alanine (Ala) via site-directed mutagenesis, introduced the mutant NAAA (NAAA-Ala^209^) into HEK293 cells by lipid-mediated transfection, confirmed the transfection by western-blot analysis (Figure S2), and then detected NAAA activity 48 hr post-transfection. The result showed that the Ala^209^ substitution in NAAA mutant significantly reduced its bioactivity comparing to wild-type NAAA (Figure. 2B), supporting the computational model outlined in Figure 2A. In addition, the pyrrolidine ring of compound **16** near the phenyl group of Tyr^151^ (average distance = 3.46 Å) incurred a hydrophobic interaction between compound **16** and the catalytic pocket of NAAA, which also contributed to the inhibition on activity.

### Stability of Compound 16

Previous reports found that current NAAA inhibitors, such as CCP and *(S)*-OOPP [3], were limited in their medicinal applications due to either inhibitory inefficiency or structural instability. To evaluate the biological and chemical stability of compound **16**, we determined the degradation products of compound **16** in different chemical environments, as well as in rat plasma. First, chemical hydrolysis was evaluated at various pHs, i.e. pH 1.0 and pH 13.0. The hydrolytic product, biphenylpropanoic acid, if any, was detected by thin layer chromatography (TLC) after 24 hr incubation with 0.1 M HCl (pH 1.0) or 0.1 M NaCl (pH 13.0). There was no detectable biphenylpropanoic acid on TLC plate (EtOAc/PE 1∶2) in either acidic medium or basic medium (Table S4). Second, to test whether compound **16** was sensitive to thermal challenge, we placed compound **16** in 80°C incubator for 24 hr. TLC plate (EtOAc/PE 1∶2) analysis showed no trace of biphenylpropanoic acid or other corresponding residuals (Table S4).

In terms of the biological stability test, rat plasma was usually chosen as reference *in vitro* model for drug stability studies [19], [20], [21]. The hydrolysis of compound **16** was studied in 80% rat plasma at 37°C physiological condition. After 8 hr and 16 hr incubation of compound **16** with rat plasma, there were 89% and 64% of compound **16** remaining in rat plasma, respectively (Table S4), indicating that compound 16 has excellent biological stability as well as chemical stability.

### Bioactivity of Compound 16 in ex-vivo

**Figure 3:**
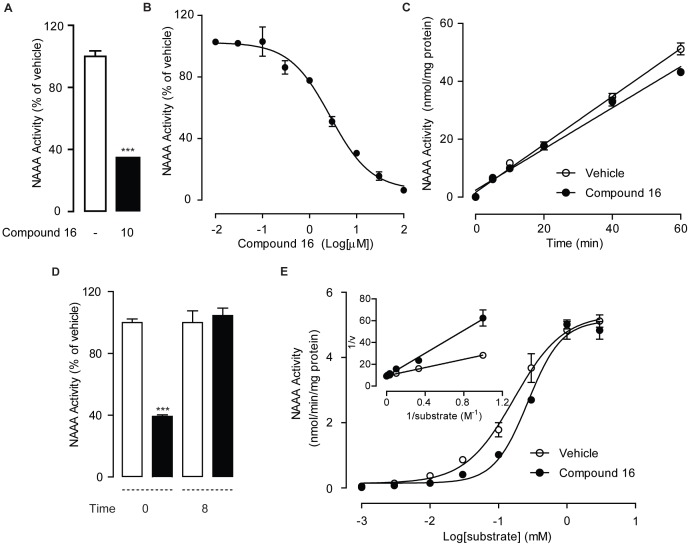
Characterization of compound 16 as a reversible and competitive NAAA inhibitor. (A) Effect of compound 16 (10 µM) on NAAA activity in HEK293 cells heterogeneously overexpressing NAAA. ***, P<0.001 vs. vehicle, n = 4. (B) Concentration-dependent inhibition of NAAA by compound 16 using NAAA recombinant protein derived from HEK293 cell heterogeneously expressing NAAA. (C) Rapid dilution NAAA assay in the presence of vehicle (1% DMSO, open circles) or compound 16 (closed circles). (D) Effect of NAAA activity in the presence of vehicle (open bars) or compound 16 (closed bars) before dialysis (0) and 8 hr after dialysis (8). ***, P<0.001 vs vehicle, n = 4; (E) Michaelis-Menten analysis of the NAAA reaction in the presence of vehicle (open circles) or compound 16 (closed circles). Insert is shown in a Lineweaver-Burk plot.

As compound **16** had demonstrated potent and selective inhibition on NAAA when activity assay was performed on NAAA protein extract, we further examined whether the same effect could be reproduced in intact cells. To test the bioactivity *ex-vivo*, HEK293 cells heterogeneously overexpressing NAAA were treated with 10 µM of compound **16** for 8 hr, and NAAA activity was measured in cells. We found that 10 µM compound **16** achieved more than 60% inhibition (p<0.001) on NAAA activity (Figure 3A) in intact cells, similar to those data obtained from NAAA protein extract (IC_50_ = 2.12±0.41 µM) (Figure 3B), indicating that compound **16** may be an ideal candidate for further *in vivo* studies.

### Compound 16 is a Reversible and Competitive NAAA Inhibitor

To further characterize the interaction between compound **16** and NAAA, we measured NAAA activity in rapid dilution assay [22], [23] and dialysis assay [24], [25]. Rapid dilution (Figure 3C) and dialysis (Figure 3D) of the compound **16**-NAAA interaction complex almost completely restored the NAAA activity. To further characterize compound 16, we performed enzyme kinetic assay using 5µM compound 16 with various substrate concentrations. Michaelis-Menten kinetic analysis revealed that compound **16** did not change the maximal catalytic velocity (V_max_) of NAAA activity (V_max_ in pmol/min/mg, vehicle, 5547±348; compound **16**, 5854±511; n = 3; p = 0.22), but it increased Michaelis-Menten constant Km (Km in µM, vehicle, 174±42; compound **16**, 328±98; p = 0.033) (Figure 3E). Based on the Km value, the dissociation constant Ki of compound 16 was calculated as 5.65 µM according to the formula as follows: Km *(inhibitor)* = Km (1+[*I*]/Ki). Taking together, these results suggested that compound **16** be a reversible and competitive NAAA inhibitor.

### Effect of Compound 16 on LPS-induced Inflammation

**Figure 4:**
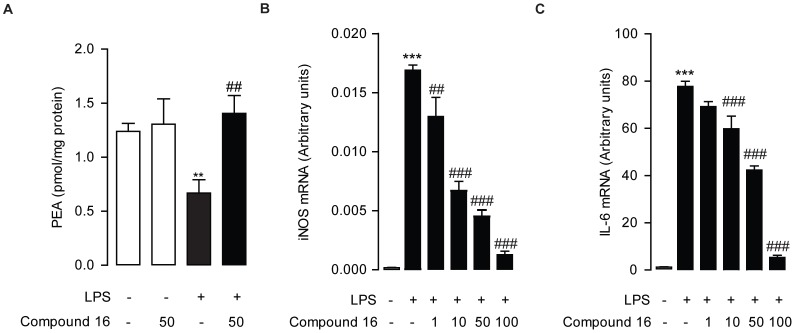
Compound 16 reduced LPS-induced inflammation. (A) Effect of compound 16 (concentrations in µM) or Vehicle on PEA levels (A), mRNA expression levels of iNOS (B) and IL-6 (C) in RAW264.7 treated with vehicle (open bars) or LPS (closed bars). vehicle, 0.1% DMSO; LPS, 0.5 µg/mL. **, P<0.01; ***, P<0.001 vs. vehicle; ##, P<0.01; ###, P<0.001 vs. LPS control, n = 5.

In order to evaluate the pharmacological effects of compound **16**, we used mouse macrophages with LPS-induced inflammation and measured cellular PEA levels by lipid analysis after the treatment of compound **16**. In RAW264.7 cells, 0.5 µg/mL LPS significantly reduced cellular PEA levels comparing to the vehicle-treated control (PEA in pmol/mg protein, vehicle, 1.23±0.07; LPS, 0.67±0.12, p = 0.0021) (Figure 4A). However, compound **16** was able to counteract the LPS-induced PEA reduction in RAW264.7 cells (in pmol/mg protein, LPS, 0.67±0.12; LPS+compound **16**, 1.41±0.17, p = 0.0037) (Figure 4A), whereas no change in PEA levels was observed when RAW264.7 cells were treated with compound **16** alone (in pmol/mg protein, vehicle, 1.23±0.07; compound **16**, 1.30±0.23, p = 0.396) (Figure 4A).

To further investigate whether the changes of cellular PEA levels mediated by compound **16** contributed to the anti-inflammatory effect, we determined the mRNA expression levels of inflammatory-response genes, including iNOS and IL-6, by quantitative PCR. In RAW264.7 cells, 0.5 µg/ml LPS elicited a drastic increase of mRNA expressions of iNOS (p<0.0001) (Figure 4B) and IL-6 (p<0.0001) (Figure 4C) and these inductions could be reversed dose-dependently by compound **16** (Figure 4B,C).

## Discussion

The present study provided new insights into the SAR study of NAAA inhibitors and discovered a novel NAAA inhibitor, 1-(2-Biphenyl-4-yl)ethyl-carbonyl pyrrolidine (compound **16**). Pharmacology studies showed that compound **16** was a reversible and competitive NAAA inhibitor, and was able to reverse LPS-induced expression of iNOS and IL-6 due to an increase of endogenous PEA levels, implying that it might be a potential anti-inflammatory agent.

To design new derivatives for SAR exploration, we utilized a three-dimensional model of NAAA built by comparative modeling, which factored in all essential features of the catalytic site of Ntn hydrolase [13] conserved in NAAA, and interpreted the critical roles of amino acid residues involved in oxyanion hole arrangement (Asn^292^), stabilization of Cys^131^ basic nitrogen (Asp^150^), and ligand recognition (Asn^209^ and Tyr^151^) [13], [16]. With this effective NAAA model, the covalent intermediates demonstrated that the amide fragment of compound **16** formed polar interaction with ligand recognition residue Asn^209^ and that the pyrrolidine head of compound **16** approached the lipophilic pocket by aligning with the aromatic ring of Tyr^151^ at a distance of 3.46 Å. We postulated that the amide bond of pyrrolidine could be the key of compound **16’**s interaction with NAAA. Based on this assumption, we mutated the ligand recognition site Asn^209^ to Ala^209^, which resulted in the loss of NAAA activity. Our computational docking data and mutagenesis studies revealed that compound **16** occupied the entrance of catalytic pocket via the interaction with Asn^209^ and Tyr^151^ so as to block the substrate recognition and entrance. Additionally, the shape-dependent lipophilic chain, the phenyl rings, of compound 16 might assist the compound-docking configuration in the binding pocket of NAAA.

Pharmacological characterization of compound **16** revealed that compound **16** inhibited NAAA through a rapid, competitive, and reversible mechanism, which was consistent with the proposed binding pocket blockage theory. In present study, we also examined whether compound **16** inhibited NAAA activity and attenuated inflammation. For this purpose, we tested compound **16** against LPS-induced inflammation in mouse macrophage RAW264.7 cells. Consistent with previous reports [3], we found that LPS lowered endogenous PEA levels, and compound **16** was able to reverse LPS-induced PEA reduction in mouse macrophages. Many studies have shown that PEA activates anti-inflammatory nuclear receptor PPAR-α [6] and that exogenous PEA exerts broad and profound anti-inflammatory effects [3], [26], [27], [28]. Therefore, we further explored whether changes of PEA levels mediated by compound **16** actually contributed to the anti-inflammatory action. Real-time PCR affirmed that compound **16** suppressed the mRNA expressions of inflammatory response elements IL-6 and iNOS that were induced by LPS. All these suggested that this novel NAAA inhibitor, compound **16**, may provide a chemical scaffold for the development of new strategies to explore and investigate NAAA’s functions and drug-like inhibitors.

## Materials and Methods

### Chemicals

All reagents were purchased from Sigma-Aldrich (Shanghai, China), seeking the highest grade commercially available.

All compounds were synthesized in our lab as described in **Text S1** and identified by NMR (Details are provided in Figure S3). Briefly, the compounds **1–6** were prepared by the amidation or esterification of palmitoyl chloride with corresponding pyrrolidine or alcohol in the presence of base. The compounds **17–20** and compounds **23–24** were prepared by the amidation of pyrrolidine with corresponding acids. The compound **21** was prepared through the reaction of isocyanate with pyrrolidine. The synthesis of isocyanate was performed via Cutius rearrangement of appropriate acylazide prepared by successive reactions of the 4–biphenylacetic acid with oxalyl chloride and sodium azide. The compound **22** was prepared by a two–step procedure starting from 4–Phenylbenzyl alcohol, which was converted to chloroformate by treating with bis(trichloromethyl) carbonate (BTC) and triethylamine; the acylation of pyrrolidine with the chloroformate produced compound **22**. The compound **25** was prepared by LiAlH_4_ reduction of compound **16**.

### Cell Culture

HEK293 cells overexpressing rat NAAA (HEK293-rNAAA) [3] and rat FAAH (HEK293-rFAAH) [29] were kind gifts from Dr. Daniele Piomelli in University of California, Irvine. The stable-overexpressing cell lines were maintained in Dulbecco’s Modified Eagle Medium (DMEM, Hyclone, Beijing, China) supplemented with 10% FBS (Gibco®, Shanghai, China) containing 0.3 mg/mL G418. Mouse macrophage cells RAW264.7 and Human Embryonic Kidney cell HEK293 cells were purchased from American Type Culture Collection (ATCC, Beijing, China) and maintained in DMEM supplemented with 10% FBS in humidified 5% CO_2_ atmosphere at 37°C. RAW264.7 cells were plated and cultured overnight until 80% confluence and then incubated with a series of compound **16** of different concentrations for 30 min before challenged by LPS.

### Protein Preparation and Enzymatic Assay

HEK293-rNAAA or HEK293-rFAAH cells were harvested, washed with PBS, sonicated in 20 mM Tris-HCl (pH 7.5) containing 0.32 M sucrose, and centrifuged at 800 × g for 15 min at 4°C. The supernatants were collected and protein concentrations were measured by BCA protein assay kit (Pierce, Shanghai, China). NAAA activity was measured by incubating 30 µg recombinant rNAAA protein with testing compound at 37°C for 30 min in 0.2 mL phosphate buffer (50 mM, pH 5.0) containing 0.1% Triton X-100, 3 mM DTT, and 25 µM heptadecenoylethanolamide as substrate. FAAH activity was measured by incubating 30 µg of rFAAH recombinant protein derived from HEK293-rFAAH cell extract at 37°C after adding 25 µM anandamide as substrate in Tris-HCl buffer (50 mM, pH 8.0) containing fatty acid-free BSA (0.05%). The reactions were terminated by adding 0.2 mL methanol containing 1 nmol heptadecanoic acid and analyzed in LC/MS.

### Lipid Extraction

Cells were harvested and homogenized in 2 mL methanol/water (1∶1, vol/vol) containing 100 pmol of [^2^H_4_]-PEA as internal standard. Lipids were extracted by 3 mL chloroform, and the organic phases were collected, dried under N_2_, and reconstituted in methanol/chloroform (3∶1, vol/vol) for LC/MS/MS analyses.

### LC/MSn

We use an Agilent 1200-LC system coupled to a 3200Q TRAP-MS detector equipped with an ESI interface (Agilent Technologies, Shanghai, China). Fatty acids were eluted through a XDB Eclipse C18 column (4.6 × 50 mm i.d., 1.8 µm Agilent Technologies) isocratically at 0.6 mL/min for 4 min with a solvent mixture of 95% methanol and 5% water, both containing 0.25% acetic acid and 5 mM ammonium acetate. The column temperature was set at 40°C. Electrospray ionization was in the negative mode, capillary voltage was −4.5 kV, and heptadecanoic acid was used as internal standard (m/z = 267 for heptadecenoic acid, m/z = 303 for arachidonic acid, and m/z = 269 for heptadecanoic acid). PEA were separated using a XDB Eclipse C18 column, and eluted with a gradient of methanol in water (from 85% to 100% methanol in 5 min, held in 100% methanol for 10 min) at a flow rate of 1 mL/min. Column temperature was kept at 25°C. Mass spectrometer (MS) detection was ionized by positive-ion atmospheric pressure chemical ionization mode (APCI^+^) and monitored in MRM mode. The parameters were set as follows: curtain gas (CUR) at 30 psi; Nebulizer pressure (GAS1) at 60 psi; and temperature at 275°C. The molecular ions were monitored at the transition of m/z 300.20-62.00 for PEA, and m/z 304.10-66.00 for [^2^H_4_]-PEA. Quantifications were calculated at chromatographic peak areas by using Analyst® version 1.4.1. software (Applied Biosystems).

### Dialysis Assay

Dialysis assay was performed using Slide-A-Lyzer Dialysis Cassettes (Pierce, Shanghai, China). Briefly, 2 mg NAAA protein was incubated with compound **16** or dimethyl sulfoxide (DMSO) in 4 mL Tris-HCl buffer (50 mM, pH 5.0) for 10 minutes at 37°C. Mixed reaction solution was loaded onto a dialysis cartridge using a syringe and incubated in Tris-HCl buffer (50 mM, pH 5.0) at 4°C for 8 h. The samples were removed from Dialysis Cassettes by syringes for NAAA enzymatic assay.

### Rapid Dilution Assay

Rapid dilution assay was performed as previously described [22]. Briefly, samples containing 100-fold concentrated rNAAA recombinant protein were pre-incubated with 10-fold the IC_50_-equivalent concentration of compound **16** or vehicle (1% DMSO) for 10 min at 37°C. Samples were then diluted 100-fold with assay buffer containing substrate to initiate reactions, and the time course of product formation was measured by LC/MS.

### Molecular Modeling

The docking of the compound **16** to NAAA active site was performed using the Glide package [3]. The 3-dimensional model of NAAA based on its alignment with conjugated bile acid hydrolase CBAH from Clostridium perfringens (CBAH, 2BJF in the Protein Data Bank) as previously described [3] was used in the molecular modeling experiment. Compounds were docked onto the NAAA binding site at a position where the substrate PEA fits into the lipophilic pocket. Bond formation between the compound and NAAA activation site was dynamically simulated.

### Mutagenesis

The cDNA encoding rat NAAA was subcloned into mammalian expression vector pcDNA3.1 (Invitrogen, Shanghai, China) following the manufacturer manual. Asparagine-to-alanine (Asn^209^-Ala^209^) point mutant was generated by site-directed mutagenesis kit (Invitrogen) and the desired mutant was subsequently confirmed by sequencing. The mutated NAAA-Ala^209^ plasmid was subsequently introduced into HEK293 cell as previously described [3].

### Real-time Quantitative PCR

Total RNA was extracted from RAW264.7 cells with TRIzol (Invitrogen) and quantified by spectrophotometer (Beckman coulter, Shanghai, China). cDNA was synthesized from 1 µg of total RNA by using ReverTra Ace qPCR RT Kit (TOYOBO, Shanghai, China) following the manufacturer’s instructions. Real-time quantitative PCR was performed in a 7300 Real Time PCR System (Applied Biosystems, Shanghai, China) and RNA levels were normalized using glyceraldehyde-3-phosphate dehydrogenase (GAPDH) as an internal standard. The primer sequences for mouse genes were as follows: (i) iNOS, forward primer (F): CCCGTCCACAGTATGTGAGGAT, reverse primer (R): CATTACCTAGAGCCGCCAGTGA; (ii) IL-6, F: AATTAAGCCTCCGACTTGTGAAG, R: CTTCCATCCAGTTGCCTTCTTG; and (iii) GAPDH, F: TTGCTGTTGAAGTCGCAGGAG, R: TGTGTCCGTCGTGGATCTGA.

## Supporting Information

Figure S1 **Effect of vehicle (open bar) and compound 16 (100 µM, closed bars) on the activity of NAAA, FAAH, MGL, and ASAH.** ***, p<0.001, one-way ANOVA, n = 3.
(PDF)

Figure S2 **The expression levels of wild-type NAAA and mutant Ala^209^-NAAA in HEK293 cells, detected by Western-blot.** Top panel, anti-Flag; Bottom panel, anti-actin. Ala209, mutant Ala^209^-NAAA transfection; NAAA, NAAA transfection; Mock, Vector transfection.
(PDF)

Figure S3 **^1^H and ^13^C NMR spectra of compounds 1–25.**
(PDF)

Table S1 **Inhibition of compounds (1)–(6) on NAAA and FAAH activities.**
(DOC)

Table S2 **Inhibition of compounds (7)–(20) on NAAA and FAAH activities.**
(DOC)

Table S3 **Inhibition of compounds (21)–(25) on NAAA and FAAH activities.**
(DOC)

Table S4 **The stability of compound 16.**
(DOC)

Text S1 **Supplementary Methods and Supplementary References.**
(DOC)
