# Supplementary figures and images for: Design and Synthesis of Potent N-Acylethanolamine-hydrolyzing Acid Amidase (NAAA) Inhibitor as Anti-Inflammatory Compounds
Source: PLoS One. 2012 Aug 20;7(8):e43023. doi: 10.1371/journal.pone.0043023 (PMC3423427; doi:10.1371/journal.pone.0043023)

Figure S1

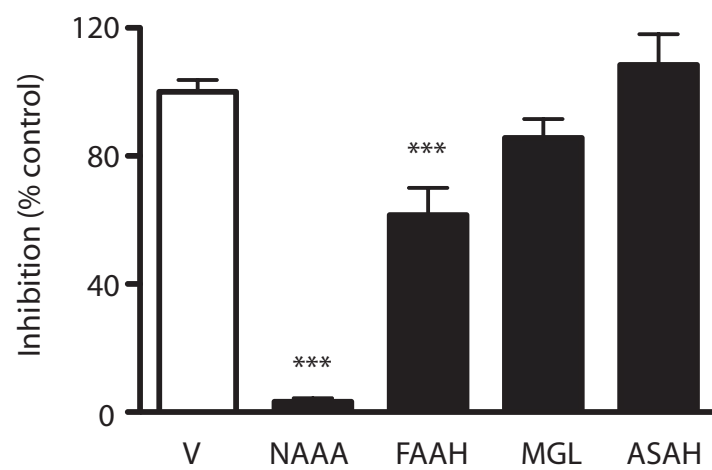

Supplement: Figure S1 — Effect of vehicle (open bar) and compound 16 (100 µM, closed bars) on the activity of NAAA, FAAH, MGL, and ASAH. ***, p<0.001, one-way ANOVA, n = 3. (PDF) [file pone.0043023.s001.pdf]

Figure S2

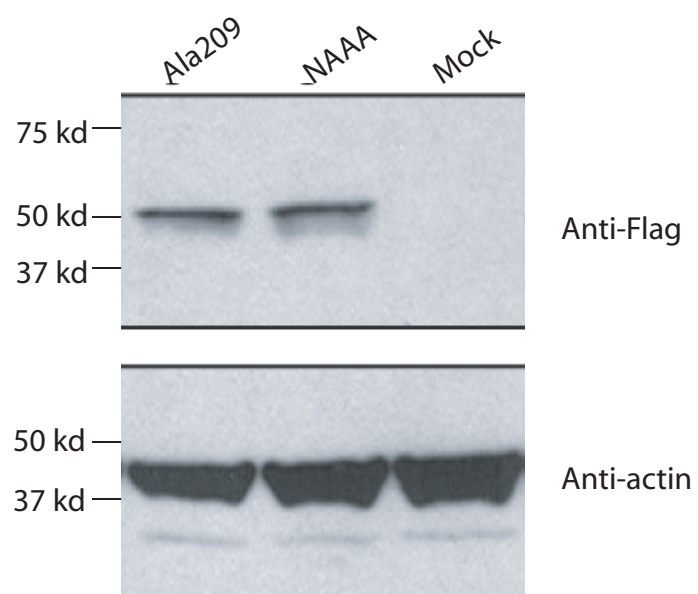

Supplement: Figure S2 — The expression levels of wild-type NAAA and mutant Ala209-NAAA in HEK293 cells, detected by Western-blot. Top panel, anti-Flag; Bottom panel, anti-actin. Ala209, mutant Ala209-NAAA transfection; NAAA, NAAA transfection; Mock, Vector transfection. (PDF) [file pone.0043023.s002.pdf]
