# Supplementary material for: Design and Synthesis of Potent N-Acylethanolamine-hydrolyzing Acid Amidase (NAAA) Inhibitor as Anti-Inflammatory Compounds
Source: PLoS One. 2012 Aug 20;7(8):e43023. doi: 10.1371/journal.pone.0043023 (PMC3423427; doi:10.1371/journal.pone.0043023)
Supplement: Figure S3 — 1H and 13C NMR spectra of compounds 1–25. (PDF) [file pone.0043023.s003.pdf]

Figure S3

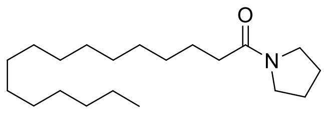

1

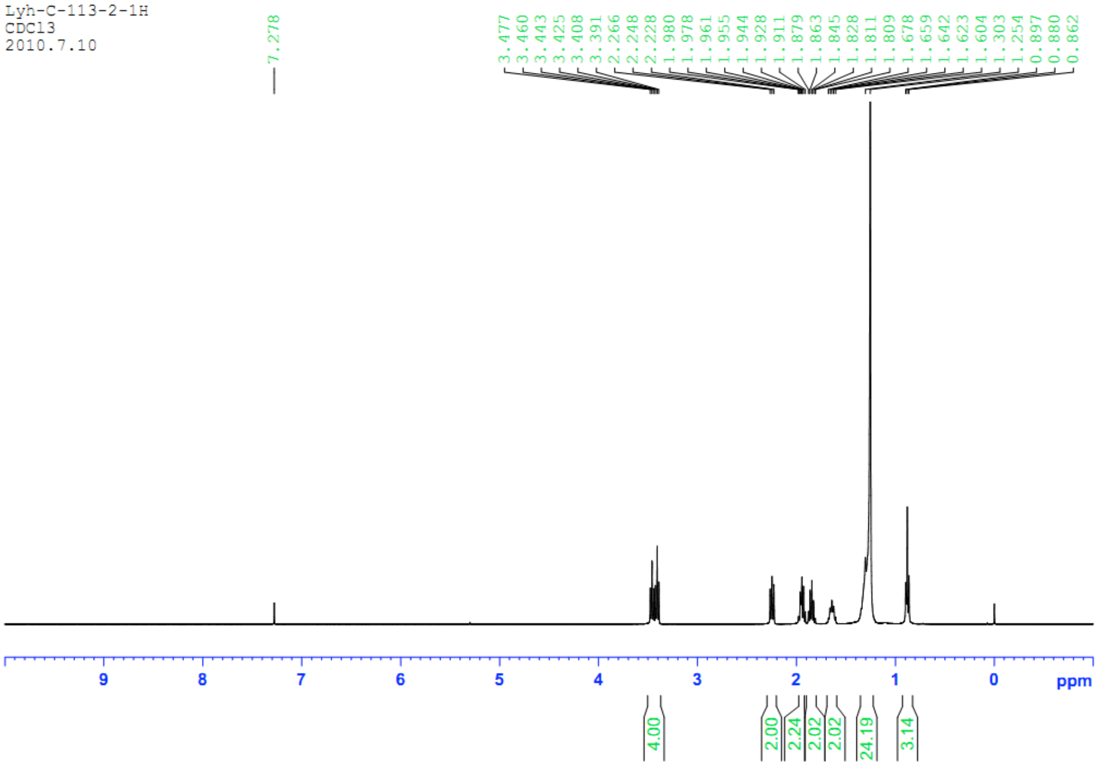

Lyh-C-113-2-1H
CDCl3
2010.7.10
7.278
3.477
3.460
3.443
3.425
3.408
3.391
2.266
2.248
2.228
1.980
1.978
1.961
1.955
1.944
1.928
1.911
1.879
1.863
1.845
1.828
1.811
1.809
1.678
1.659
1.642
1.623
1.604
1.303
1.254
0.897
0.880
0.862
9
8
7
6
5
4
3
2
1
0
ppm
4.00
2.00
2.24
2.02
2.02
24.19
3.14

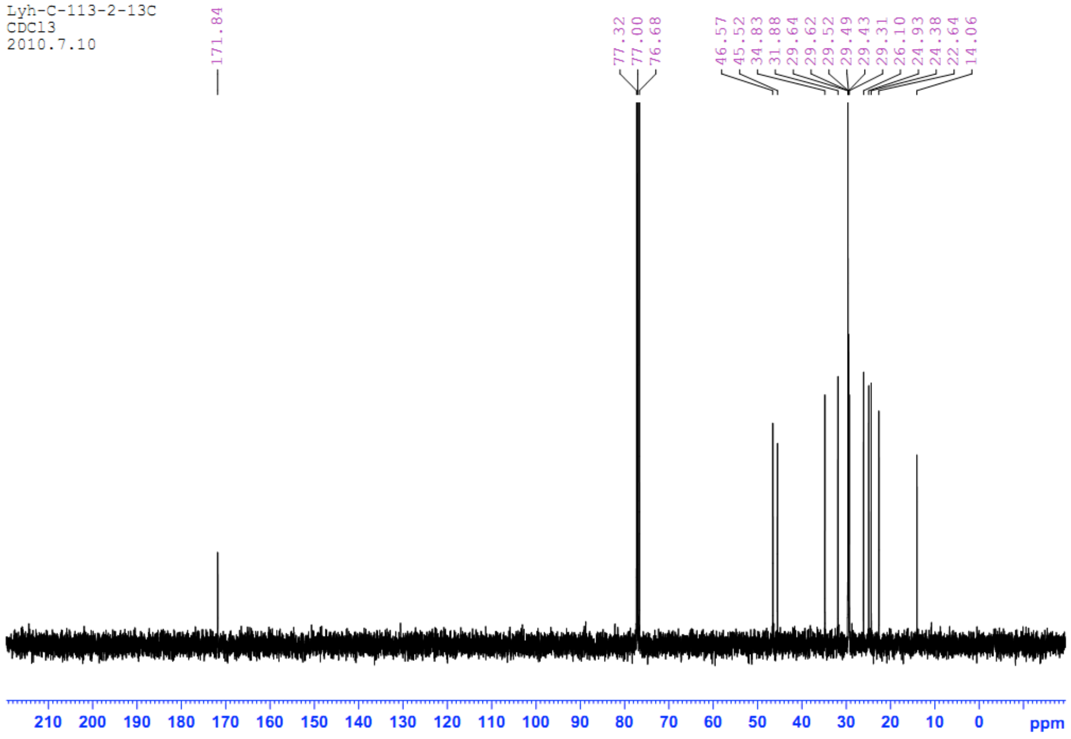

Lyh-C-113-2-13C
CDCl3
2010.7.10
171.84
77.32
77.00
76.68
46.57
45.52
34.83
31.88
29.64
29.62
29.52
29.49
29.43
29.31
26.10
24.93
24.38
22.64
14.06
210
200
190
180
170
160
150
140
130
120
110
100
90
80
70
60
50
40
30
20
10
0
ppm

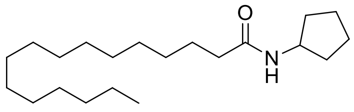

2

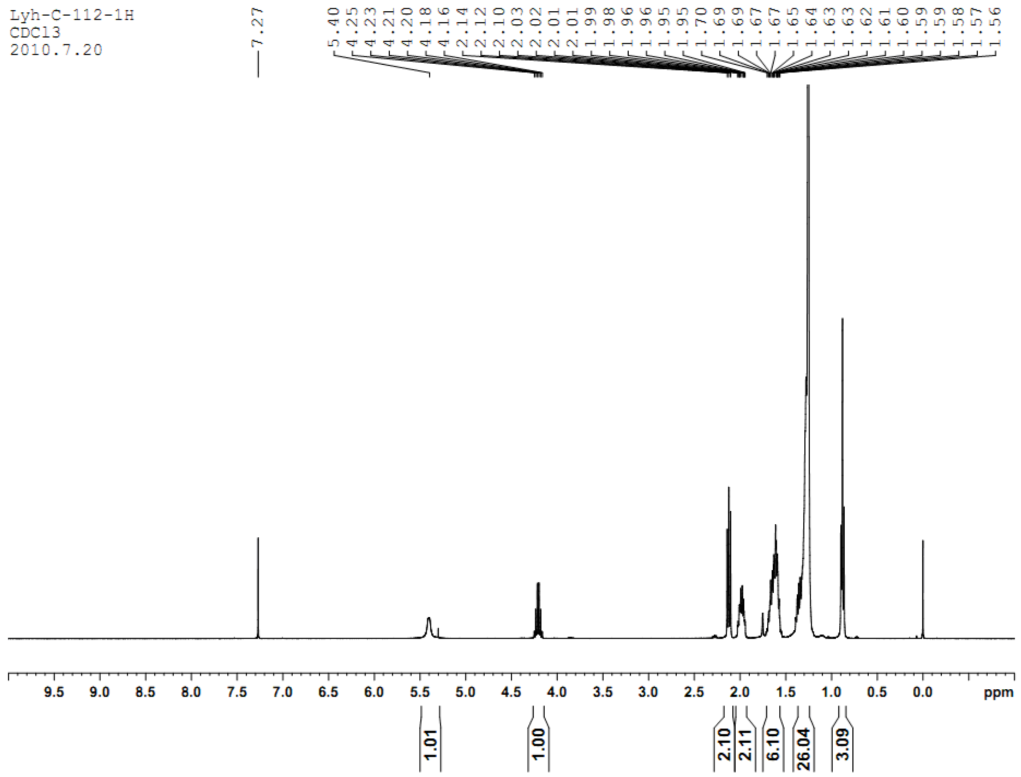
Lyh-C-112-1H
CDCl3
2010.7.20
7.27
5.40
4.25
4.23
4.21
4.20
4.18
4.16
2.14
2.12
2.10
2.03
2.02
2.01
1.99
1.98
1.96
1.96
1.95
1.95
1.70
1.69
1.69
1.67
1.67
1.65
1.64
1.63
1.63
1.62
1.61
1.60
1.59
1.59
1.58
1.57
1.56
9.5 9.0 8.5 8.0 7.5 7.0 6.5 6.0 5.5 5.0 4.5 4.0 3.5 3.0 2.5 2.0 1.5 1.0 0.5 0.0 ppm
1.01
1.00
2.10
2.11
6.10
26.04
3.09

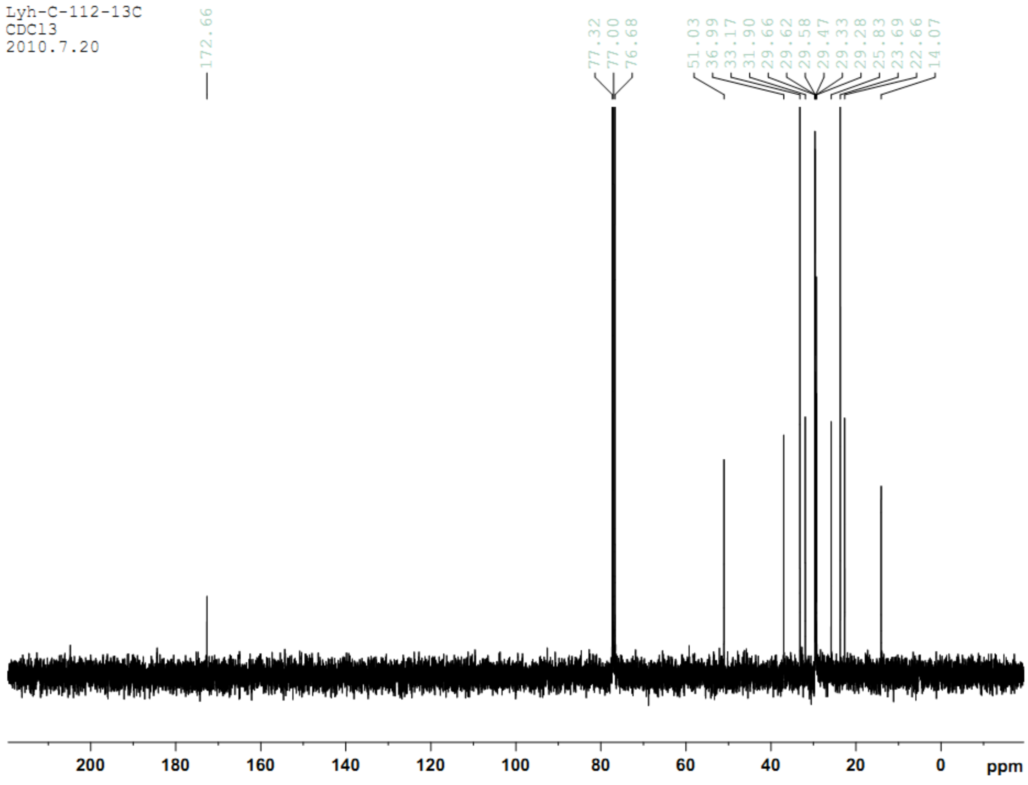
Lyh-C-112-13C
CDCl3
2010.7.20
172.66
77.32
77.00
76.68
51.03
36.99
33.17
31.90
29.66
29.62
29.58
29.47
29.33
29.28
25.83
23.69
22.66
14.07
200 180 160 140 120 100 80 60 40 20 0 ppm

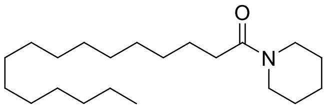
O
N

3

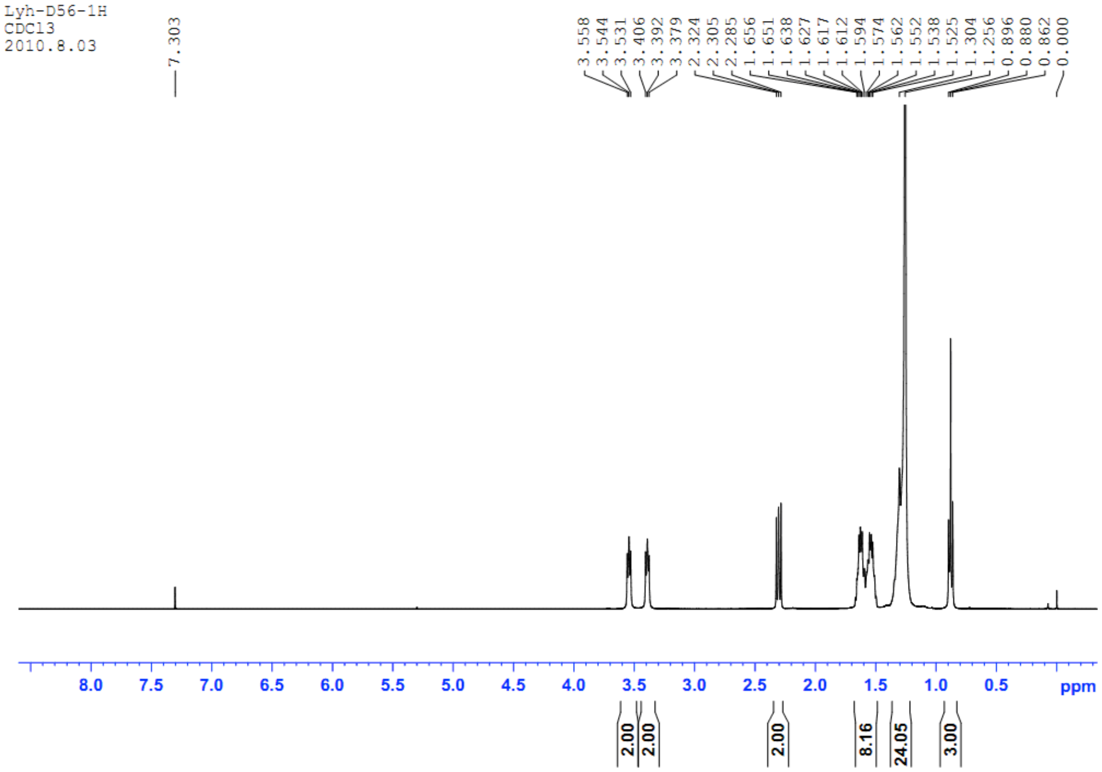
Lyh-D56-1H
CDCl3
2010.8.03
7.303
3.558
3.544
3.531
3.406
3.392
3.379
2.324
2.305
2.285
1.656
1.651
1.638
1.627
1.617
1.612
1.594
1.574
1.562
1.552
1.538
1.525
1.304
1.256
0.896
0.880
0.862
0.000
8.0 7.5 7.0 6.5 6.0 5.5 5.0 4.5 4.0 3.5 3.0 2.5 2.0 1.5 1.0 0.5 ppm
2.00
2.00
2.00
8.16
24.05
3.00

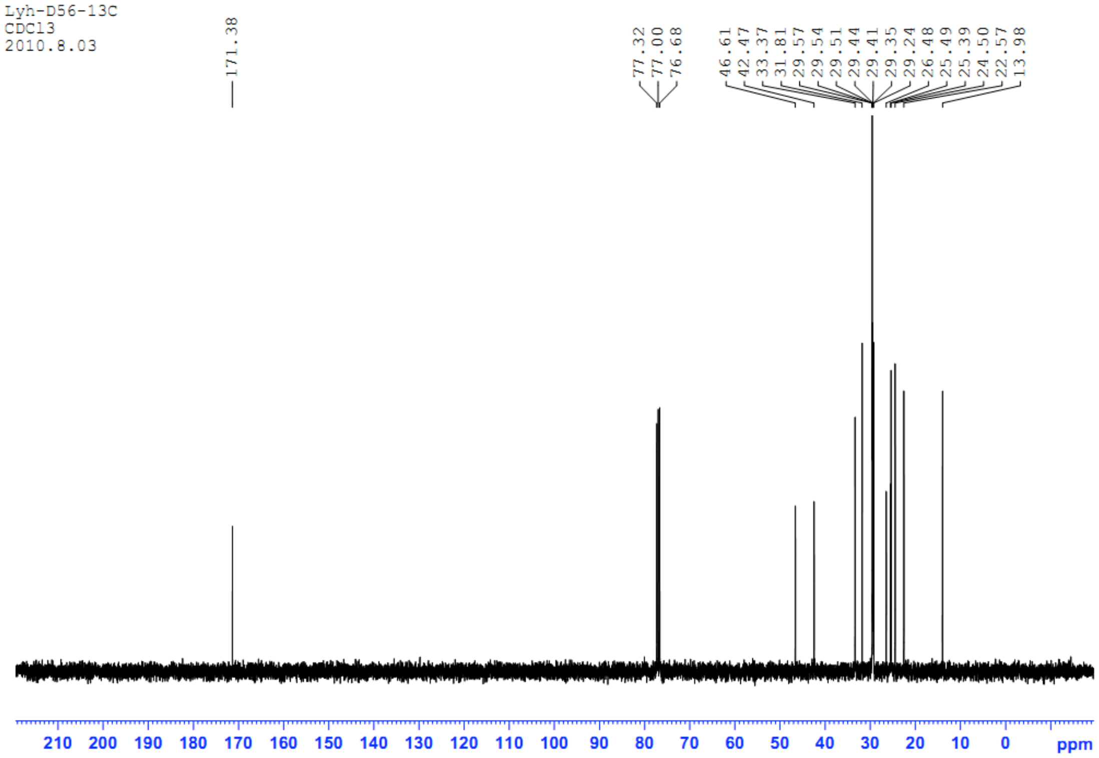
Lyh-D56-13C
CDCl3
2010.8.03
171.38
77.32
77.00
76.68
46.61
42.47
33.37
31.81
29.57
29.54
29.51
29.44
29.41
29.35
29.24
26.48
25.49
25.39
24.50
22.57
13.98
210 200 190 180 170 160 150 140 130 120 110 100 90 80 70 60 50 40 30 20 10 0 ppm

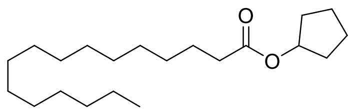
O
O

4

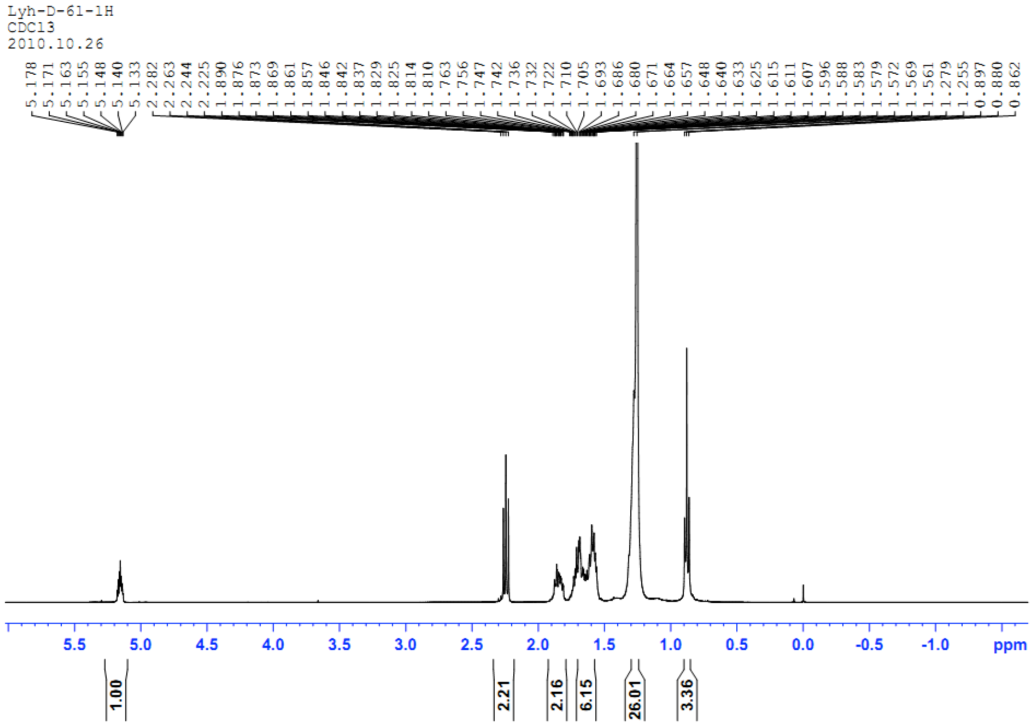
Lyh-D-61-1H
CDCl3
2010.10.26
5.178 5.171 5.163 5.155 5.148 5.140 5.133 2.282 2.263 2.244 2.225 1.890 1.876 1.873 1.869 1.861 1.857 1.846 1.842 1.837 1.829 1.825 1.814 1.810 1.763 1.756 1.747 1.742 1.736 1.732 1.722 1.710 1.705 1.693 1.686 1.680 1.671 1.664 1.657 1.648 1.640 1.633 1.625 1.615 1.611 1.607 1.596 1.588 1.583 1.579 1.572 1.569 1.561 1.279 1.255 0.897 0.880 0.862
5.5 5.0 4.5 4.0 3.5 3.0 2.5 2.0 1.5 1.0 0.5 0.0 -0.5 -1.0 ppm
1.00
2.21
2.16
6.15
26.01
3.36

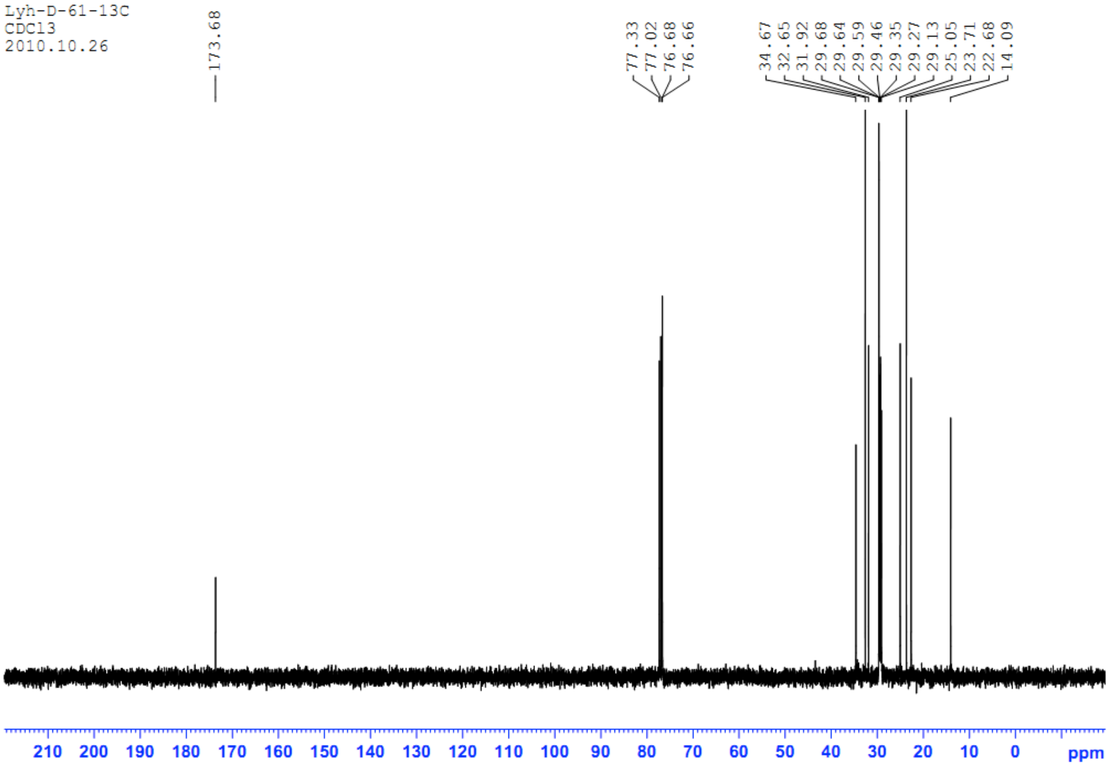
Lyh-D-61-13C
CDCl3
2010.10.26
173.68
77.33 77.02 76.68 76.66
34.67 32.65 31.92 29.68 29.64 29.59 29.46 29.35 29.27 29.13 25.05 23.71 22.68 14.09
210 200 190 180 170 160 150 140 130 120 110 100 90 80 70 60 50 40 30 20 10 0 ppm

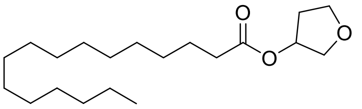

5

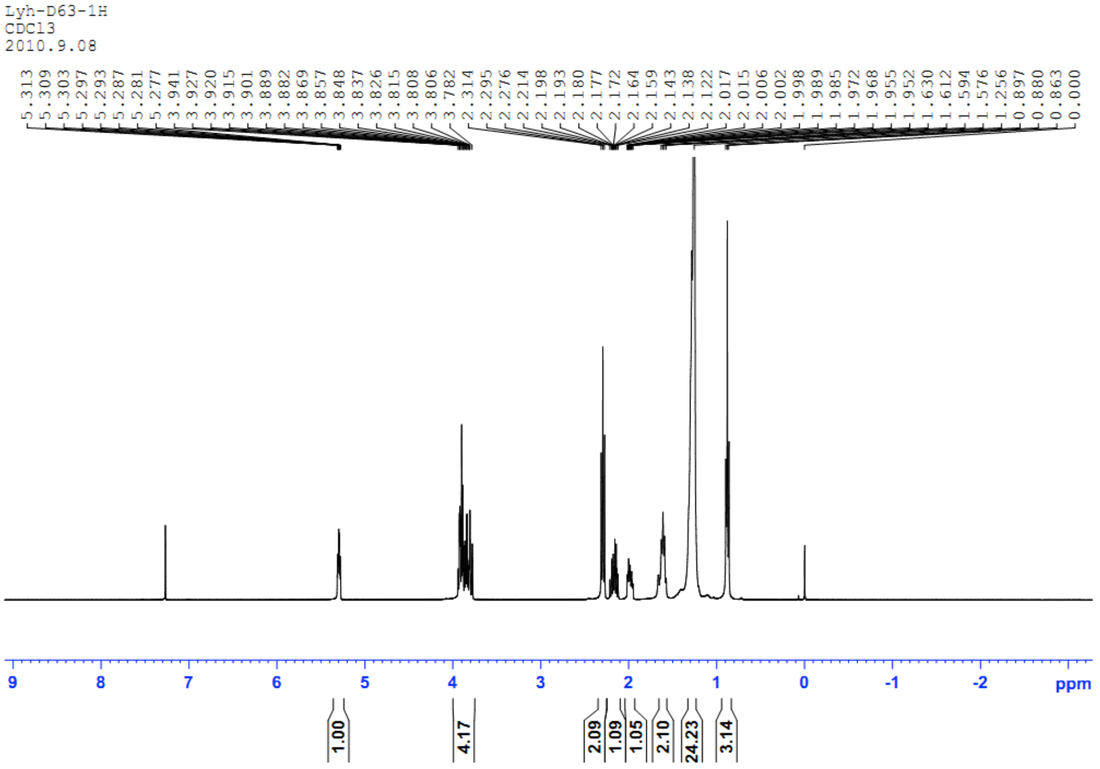
Lyh-D63-1H
CDCl3
2010.9.08
5.313
5.309
5.303
5.297
5.293
5.287
5.281
5.277
3.941
3.927
3.920
3.915
3.901
3.889
3.882
3.869
3.857
3.848
3.837
3.826
3.815
3.808
3.806
3.782
2.314
2.295
2.276
2.214
2.198
2.193
2.180
2.177
2.172
2.164
2.159
2.143
2.138
2.122
2.017
2.015
2.006
2.002
1.998
1.989
1.985
1.972
1.968
1.955
1.952
1.630
1.612
1.594
1.576
1.256
0.897
0.880
0.863
0.000
1.00
4.17
2.09
1.09
1.05
2.10
24.23
3.14
ppm

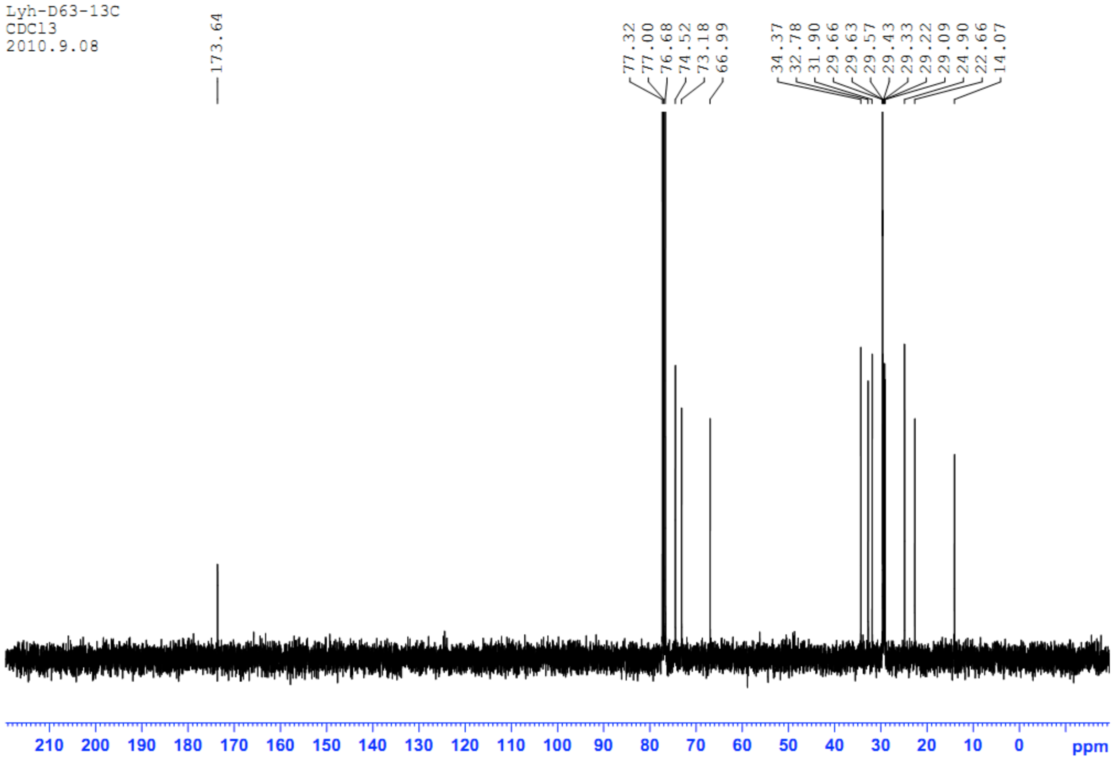
Lyh-D63-13C
CDCl3
2010.9.08
173.64
77.32
77.00
76.68
74.52
73.18
66.99
34.37
32.78
31.90
29.66
29.63
29.57
29.43
29.33
29.22
29.09
24.90
22.66
14.07
ppm

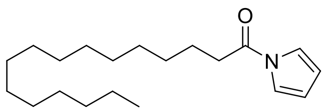
O
N

6

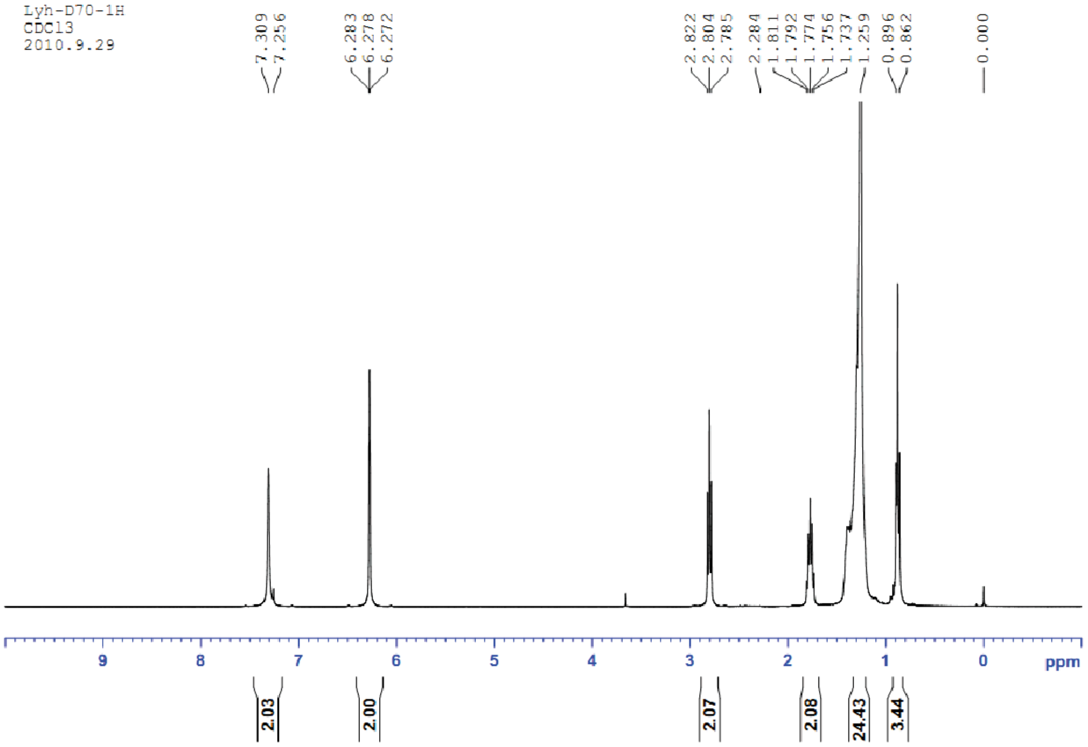
Lyh-D70-1H
CDCl3
2010.9.29
7.309
7.256
6.283
6.278
6.272
2.822
2.804
2.785
2.284
1.811
1.792
1.774
1.756
1.737
1.259
0.896
0.862
0.000
9
8
7
6
5
4
3
2
1
0
ppm
2.03
2.00
2.07
2.08
24.43
3.44

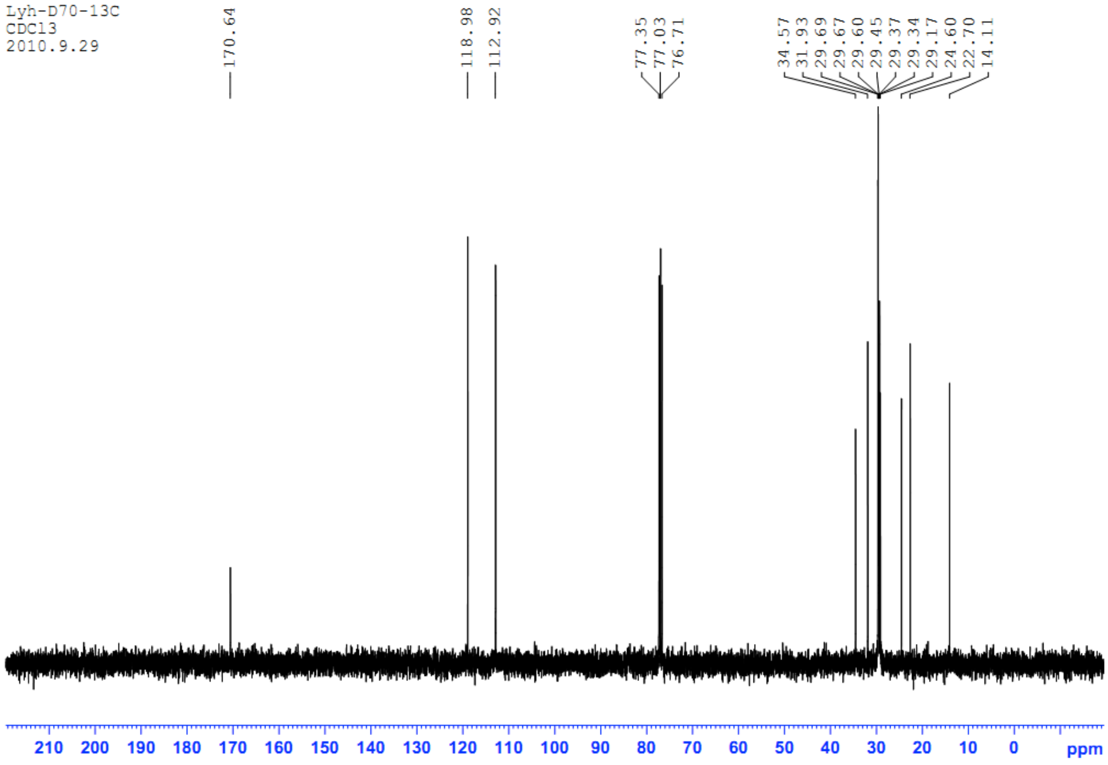
Lyh-D70-13C
CDCl3
2010.9.29
170.64
118.98
112.92
77.35
77.03
76.71
34.57
31.93
29.69
29.67
29.60
29.45
29.37
29.34
29.17
24.60
22.70
14.11
210
200
190
180
170
160
150
140
130
120
110
100
90
80
70
60
50
40
30
20
10
0
ppm

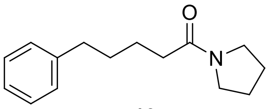

10

Lyh-D-101-1H
CDCl3
2010.11.18

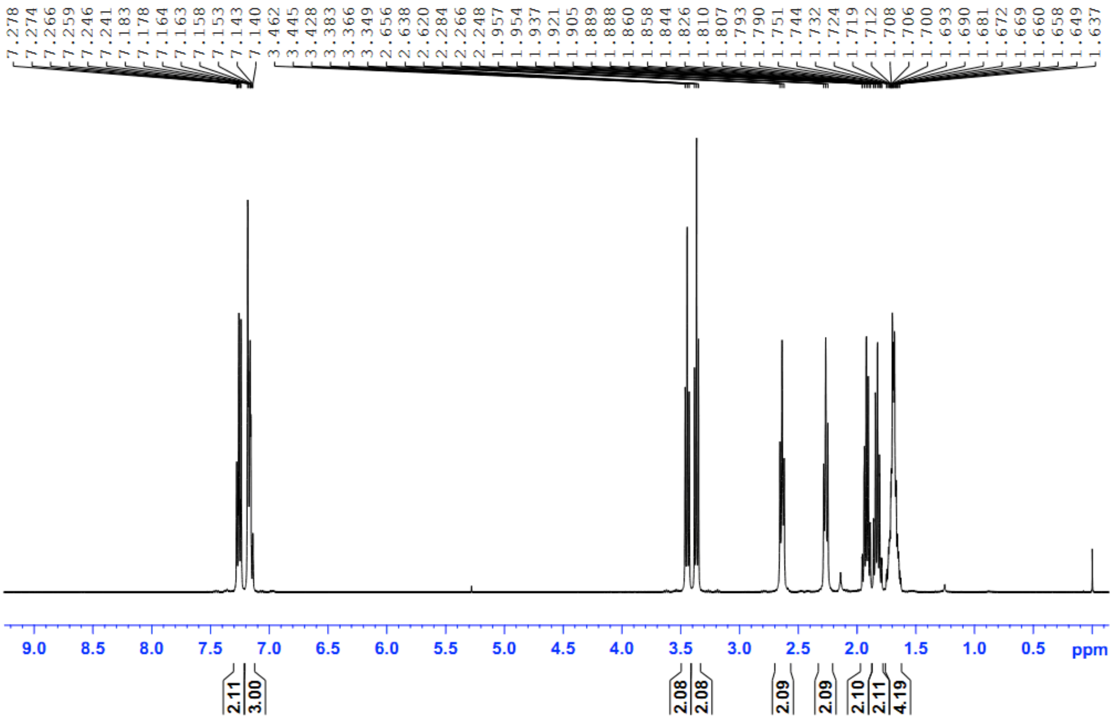

Lyh-D-101-13C
CDCl3
2010.11.18

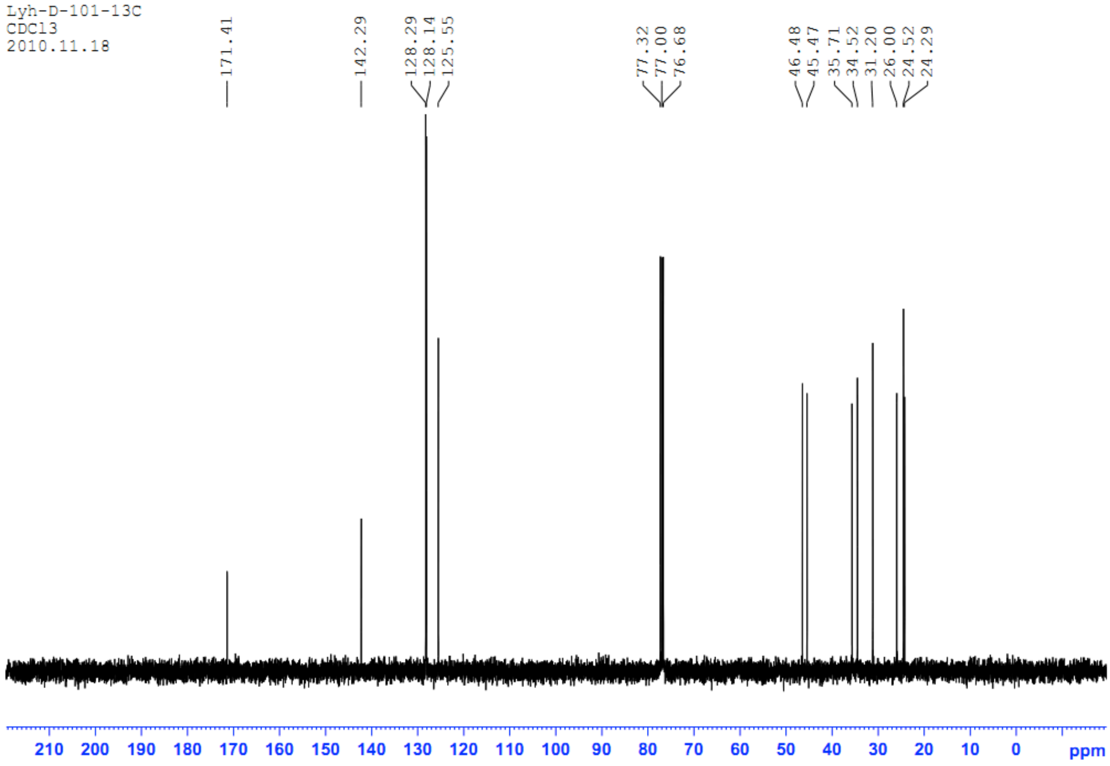

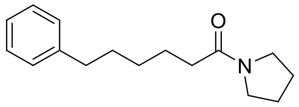
O
N

11

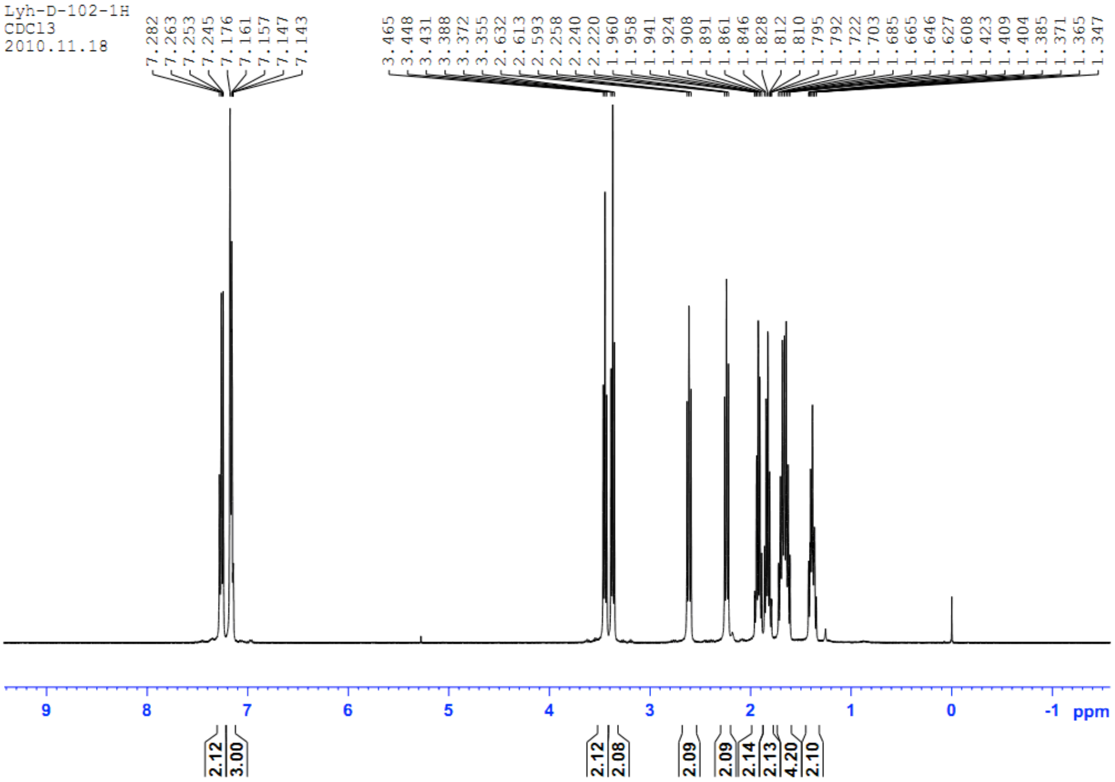
Lyh-D-102-1H
CDCl3
2010.11.18
7.282
7.263
7.253
7.245
7.176
7.161
7.157
7.147
7.143
3.465
3.448
3.431
3.388
3.372
3.355
2.632
2.613
2.593
2.258
2.240
2.220
1.960
1.958
1.941
1.924
1.908
1.891
1.861
1.846
1.828
1.812
1.810
1.795
1.792
1.722
1.703
1.685
1.665
1.646
1.627
1.608
1.423
1.409
1.404
1.385
1.371
1.365
1.347
9
8
7
6
5
4
3
2
1
0
-1
ppm
2.12
3.00
2.12
2.08
2.09
2.09
2.14
2.13
4.20
2.10

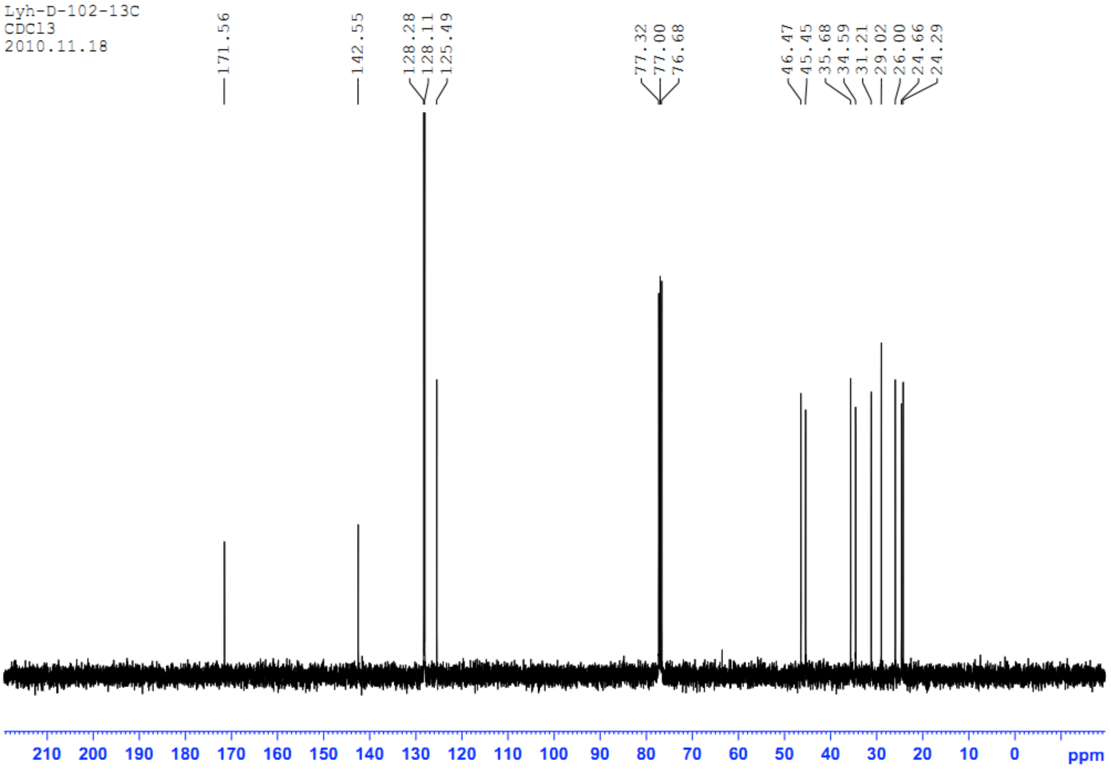
Lyh-D-102-13C
CDCl3
2010.11.18
171.56
142.55
128.28
128.11
125.49
77.32
77.00
76.68
46.47
45.45
35.68
34.59
31.21
29.02
26.00
24.66
24.29
210
200
190
180
170
160
150
140
130
120
110
100
90
80
70
60
50
40
30
20
10
0
ppm

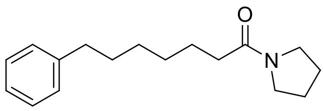

12

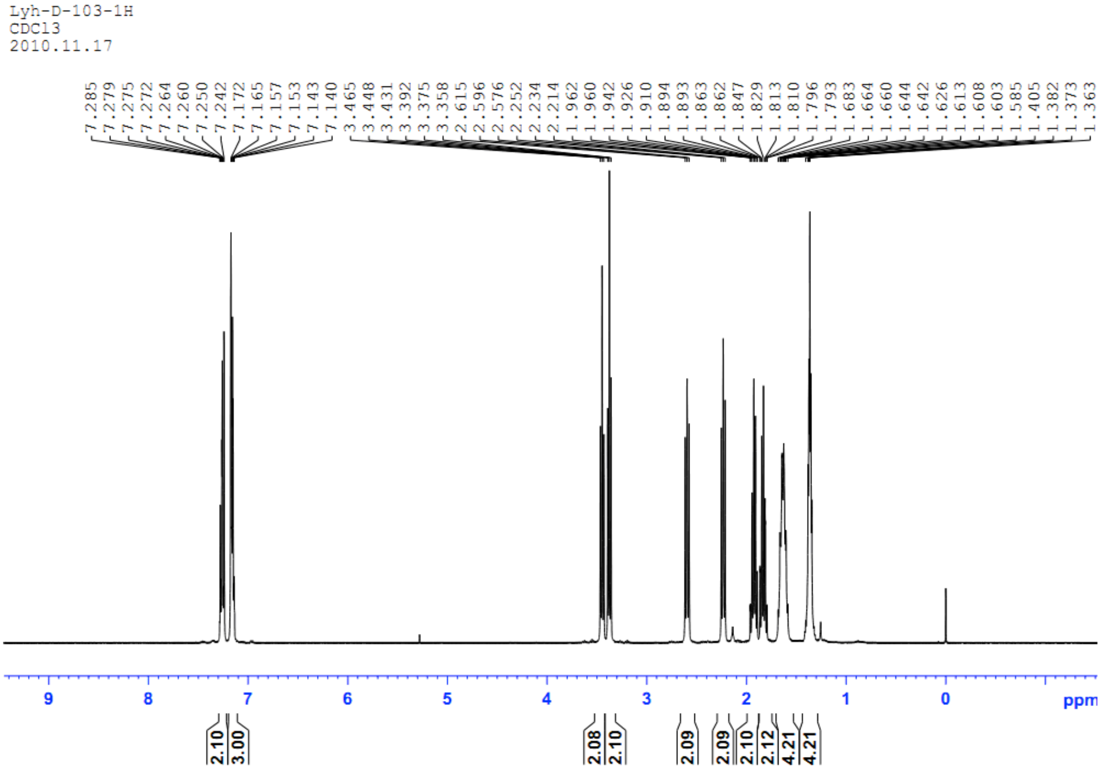
Lyh-D-103-1H
CDCl3
2010.11.17
7.285 7.279 7.275 7.272 7.264 7.260 7.250 7.242 7.172 7.165 7.157 7.153 7.143 7.140 3.465 3.448 3.431 3.392 3.375 3.358 2.615 2.596 2.576 2.252 2.234 2.214 1.962 1.960 1.942 1.926 1.910 1.894 1.893 1.863 1.862 1.847 1.829 1.813 1.810 1.796 1.793 1.683 1.664 1.660 1.644 1.642 1.626 1.613 1.608 1.603 1.585 1.405 1.382 1.373 1.363
9 8 7 6 5 4 3 2 1 0 ppm
2.10 3.00 2.08 2.10 2.09 2.09 2.10 2.12 4.21 4.21

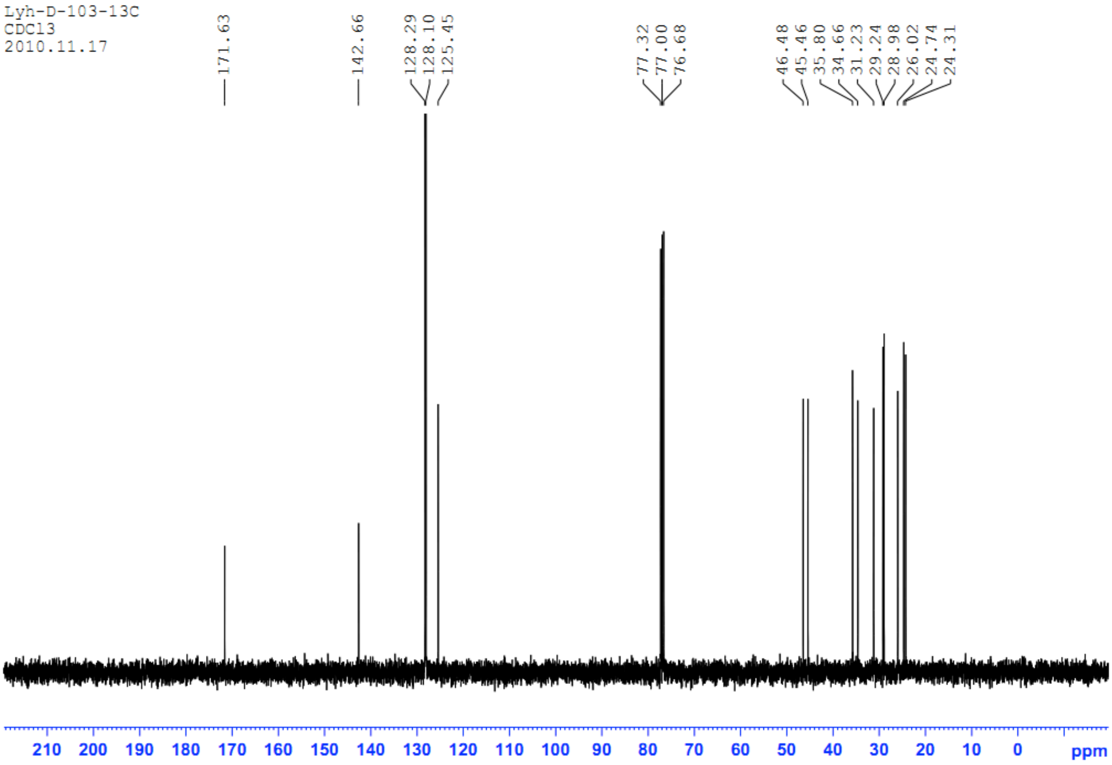
Lyh-D-103-13C
CDCl3
2010.11.17
171.63 142.66 128.29 128.10 125.45 77.32 77.00 76.68 46.48 45.46 35.80 34.66 31.23 29.24 28.98 26.02 24.74 24.31
210 200 190 180 170 160 150 140 130 120 110 100 90 80 70 60 50 40 30 20 10 0 ppm

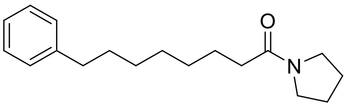

13

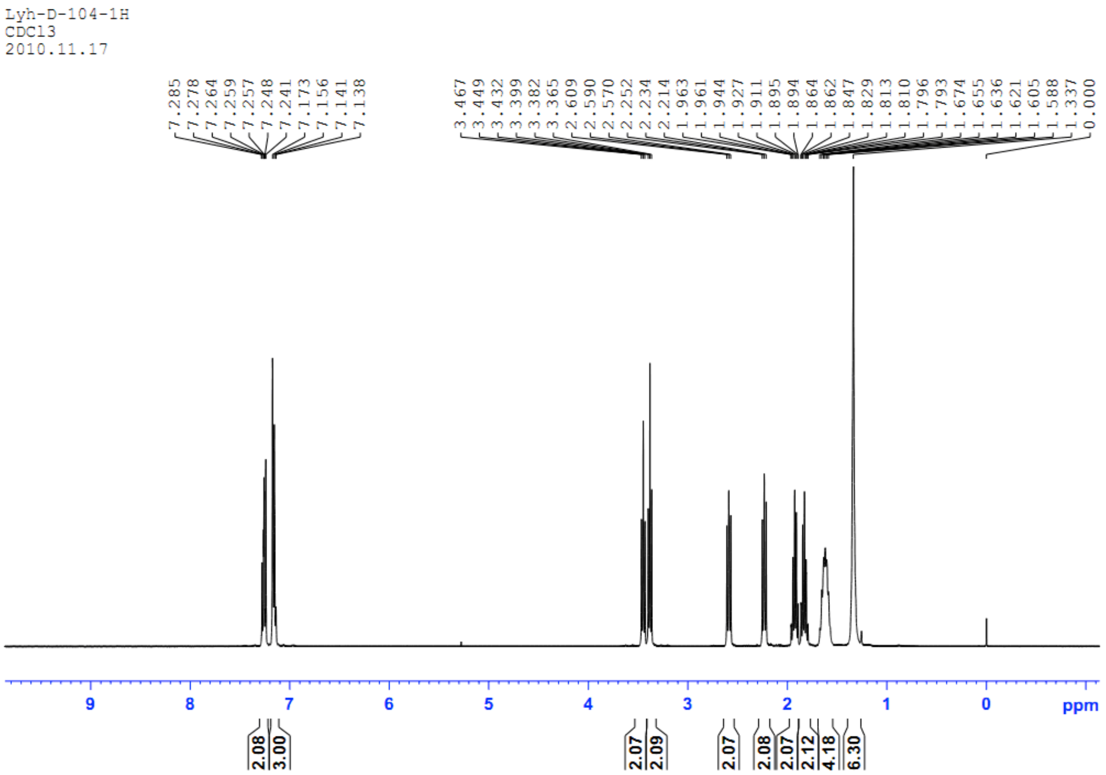
Lyh-D-104-1H
CDCl3
2010.11.17
7.285 7.278 7.264 7.259 7.257 7.248 7.241 7.173 7.156 7.141 7.138
3.467 3.449 3.432 3.399 3.382 3.365 2.609 2.590 2.570 2.252 2.234 2.214 1.963 1.961 1.944 1.927 1.911 1.895 1.894 1.864 1.862 1.847 1.829 1.813 1.810 1.796 1.793 1.674 1.655 1.636 1.621 1.605 1.588 1.337 0.000
9 8 7 6 5 4 3 2 1 0 ppm
2.08 3.00 2.07 2.09 2.07 2.08 2.07 2.12 4.18 6.30

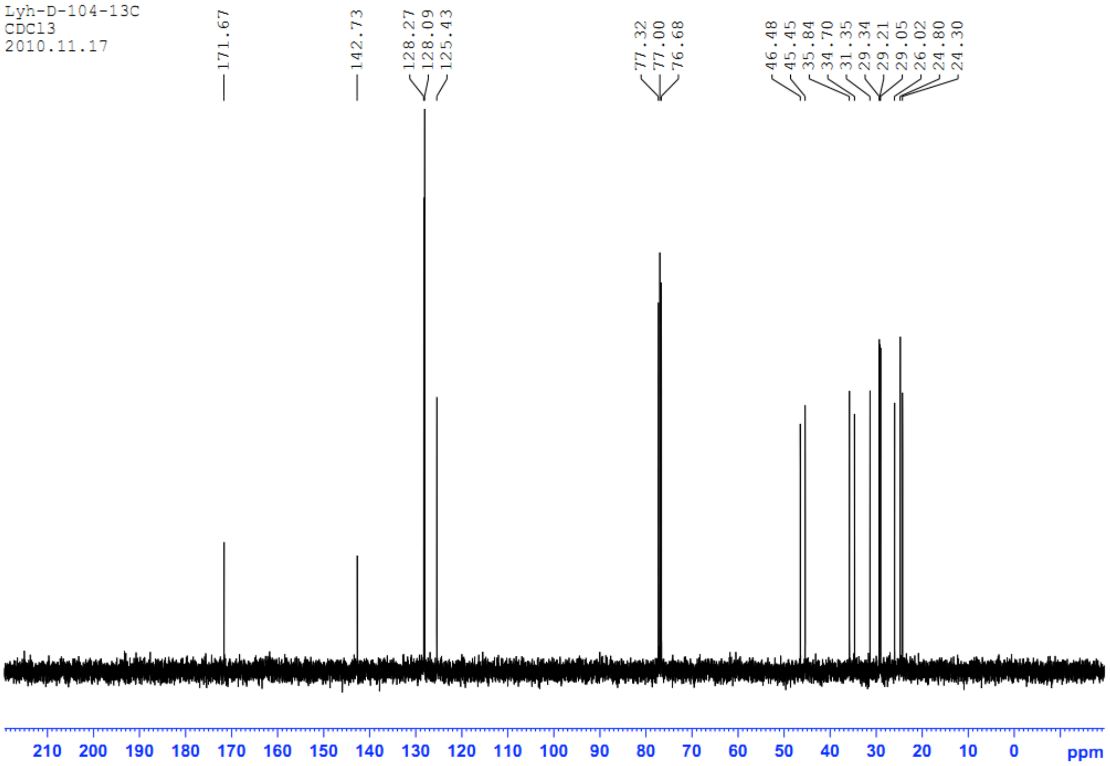
Lyh-D-104-13C
CDCl3
2010.11.17
171.67
142.73
128.27 128.09 125.43
77.32 77.00 76.68
46.48 45.45 35.84 34.70 31.35 29.34 29.21 29.05 26.02 24.80 24.30
210 200 190 180 170 160 150 140 130 120 110 100 90 80 70 60 50 40 30 20 10 0 ppm

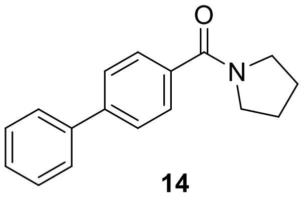

14

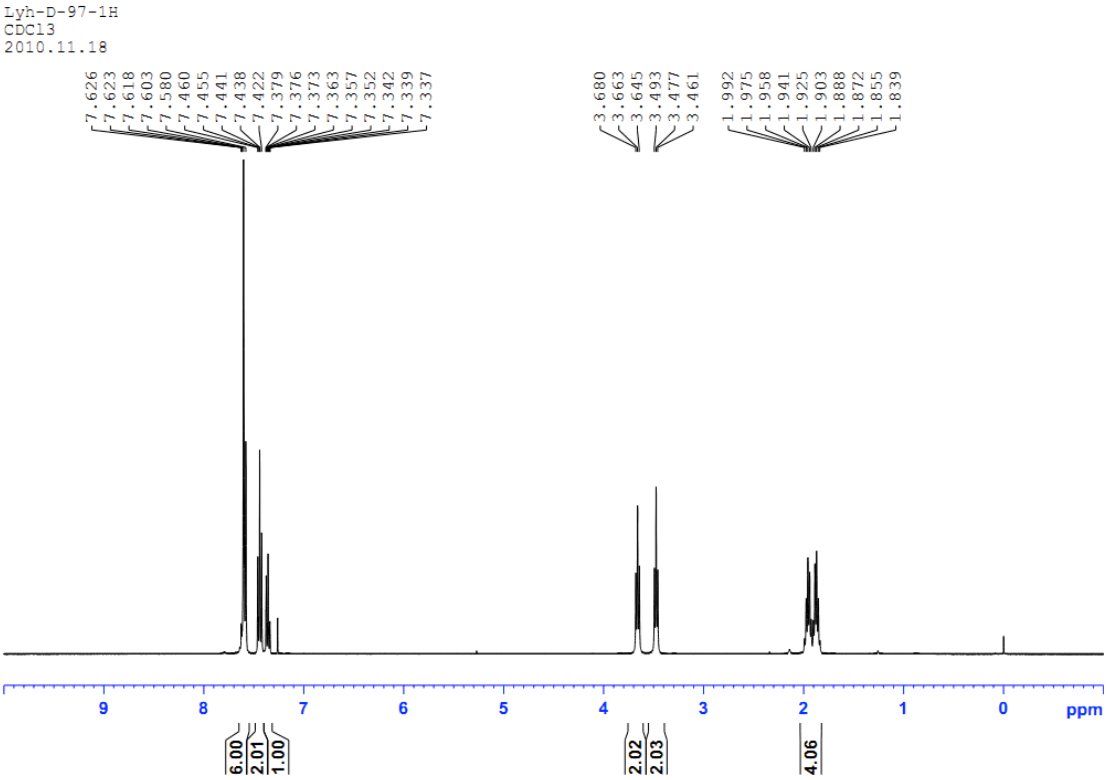
Lyh-D-97-1H
CDCl3
2010.11.18
7.626
7.623
7.618
7.603
7.580
7.460
7.455
7.441
7.438
7.422
7.379
7.376
7.373
7.363
7.357
7.352
7.342
7.339
7.337
3.680
3.663
3.645
3.493
3.477
3.461
1.992
1.975
1.958
1.941
1.925
1.903
1.888
1.872
1.855
1.839
9
8
7
6
5
4
3
2
1
0
ppm
6.00
2.01
1.00
2.02
2.03
4.06

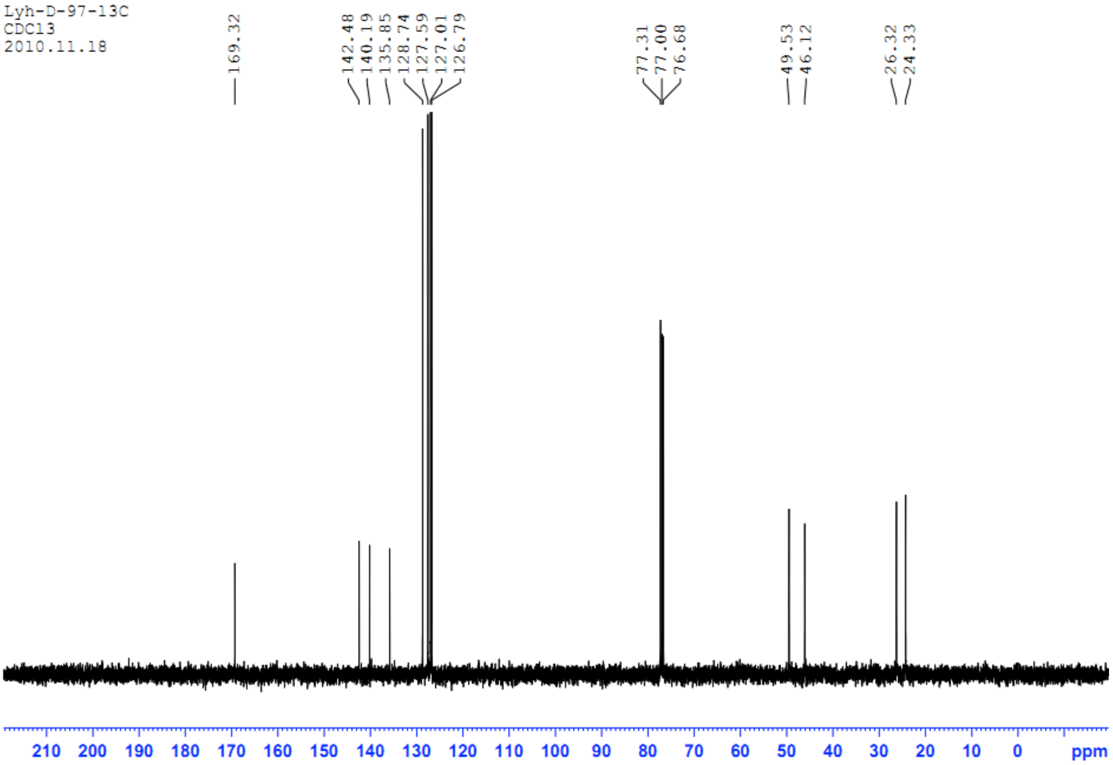
Lyh-D-97-13C
CDCl3
2010.11.18
169.32
142.48
140.19
135.85
128.74
127.59
127.01
126.79
77.31
77.00
76.68
49.53
46.12
26.32
24.33
210
200
190
180
170
160
150
140
130
120
110
100
90
80
70
60
50
40
30
20
10
0
ppm

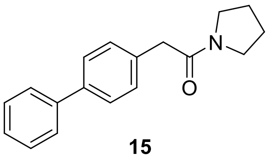

15

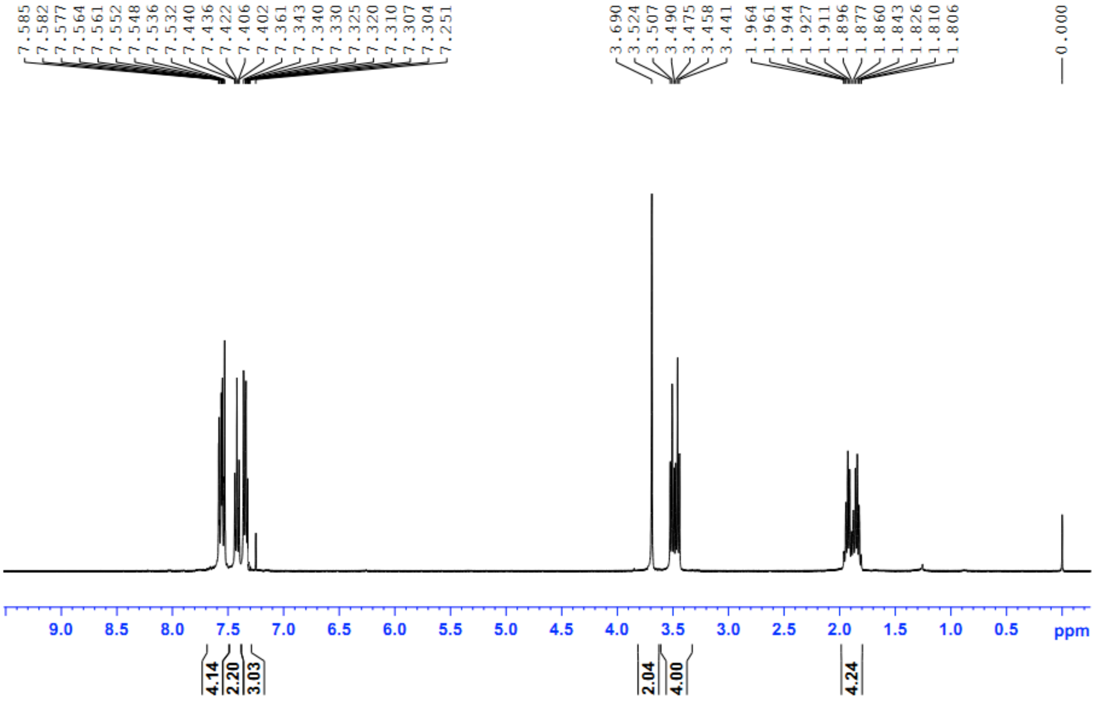

Lyh-E-9-1H
CDCl3
2010.12.01
7.585 7.582 7.577 7.564 7.561 7.552 7.548 7.536 7.532 7.440 7.436 7.422 7.406 7.402 7.361 7.343 7.340 7.330 7.325 7.320 7.310 7.307 7.304 7.251
3.690 3.524 3.507 3.490 3.475 3.458 3.441
1.964 1.961 1.944 1.927 1.911 1.896 1.877 1.860 1.843 1.826 1.810 1.806
0.000
9.0 8.5 8.0 7.5 7.0 6.5 6.0 5.5 5.0 4.5 4.0 3.5 3.0 2.5 2.0 1.5 1.0 0.5 ppm
4.14 2.20 3.03 2.04 4.00 4.24

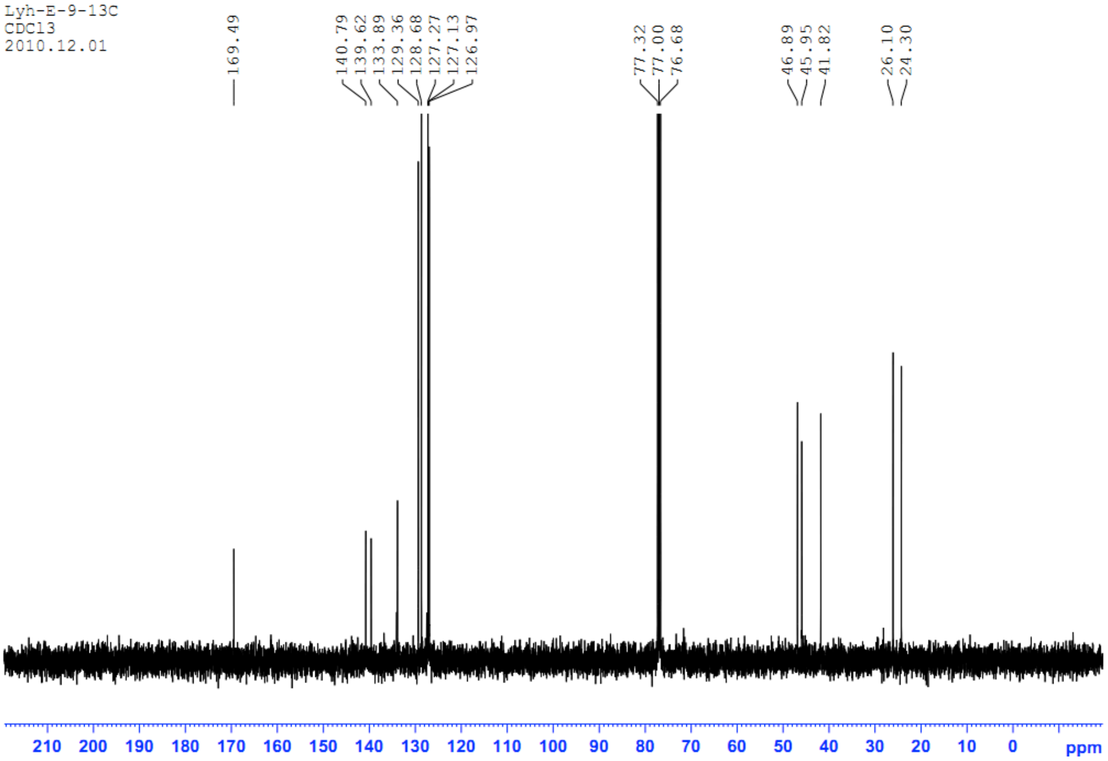

Lyh-E-9-13C
CDCl3
2010.12.01
169.49
140.79 139.62 133.89 129.36 128.68 127.27 127.13 126.97
77.32 77.00 76.68
46.89 45.95 41.82
26.10 24.30
210 200 190 180 170 160 150 140 130 120 110 100 90 80 70 60 50 40 30 20 10 0 ppm

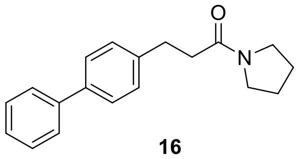

16

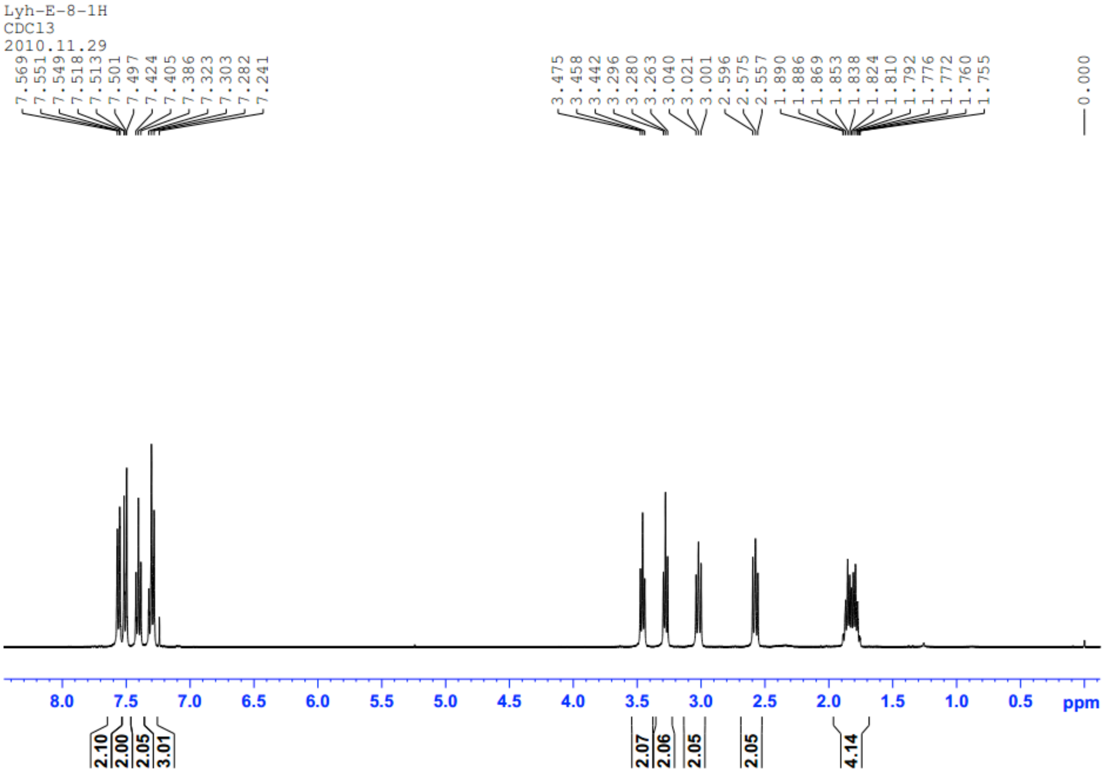
Lyh-E-8-1H
CDCl3
2010.11.29
7.569
7.551
7.549
7.518
7.513
7.501
7.497
7.424
7.405
7.386
7.323
7.303
7.282
7.241
3.475
3.458
3.442
3.296
3.280
3.263
3.040
3.021
3.001
2.596
2.575
2.557
1.890
1.886
1.869
1.853
1.838
1.824
1.810
1.792
1.776
1.772
1.760
1.755
0.000
8.0
7.5
7.0
6.5
6.0
5.5
5.0
4.5
4.0
3.5
3.0
2.5
2.0
1.5
1.0
0.5
ppm
2.10
2.00
2.05
3.01
2.07
2.06
2.05
2.05
4.14

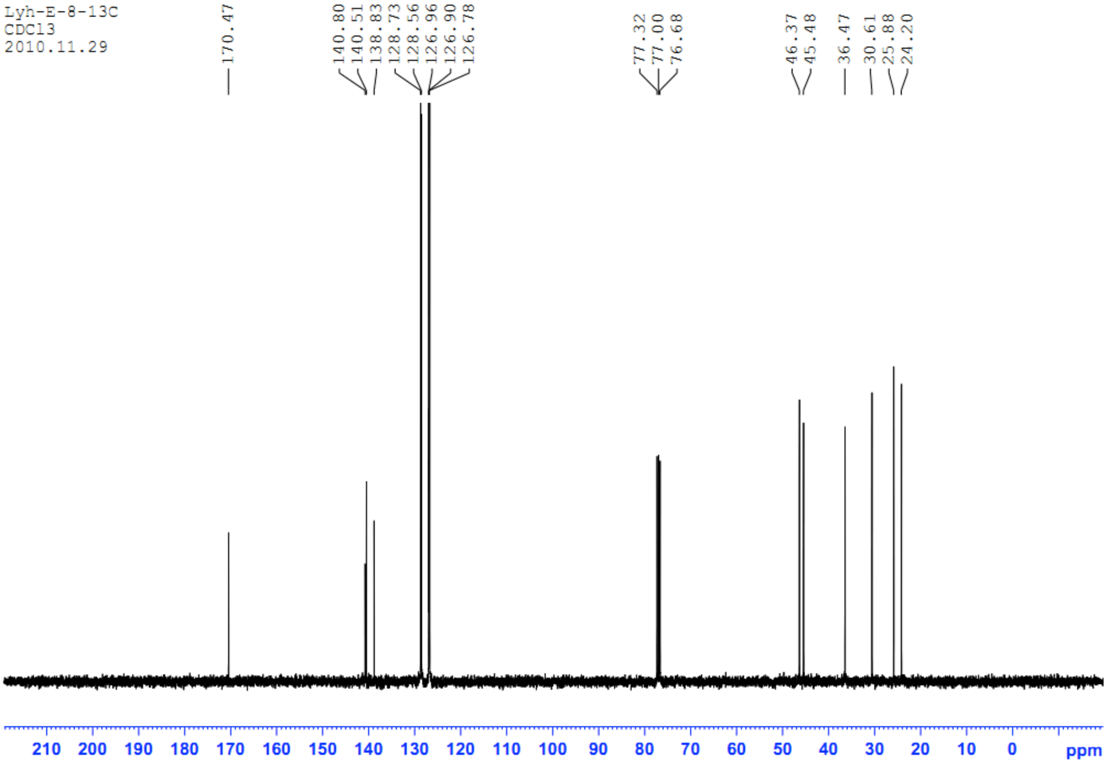
Lyh-E-8-13C
CDCl3
2010.11.29
170.47
140.80
140.51
138.83
128.73
128.56
126.96
126.90
126.78
77.32
77.00
76.68
46.37
45.48
36.47
30.61
25.88
24.20
210
200
190
180
170
160
150
140
130
120
110
100
90
80
70
60
50
40
30
20
10
0
ppm

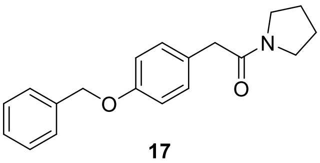

17

Lyh-E-12-1H
CDCl3
2010.12.05

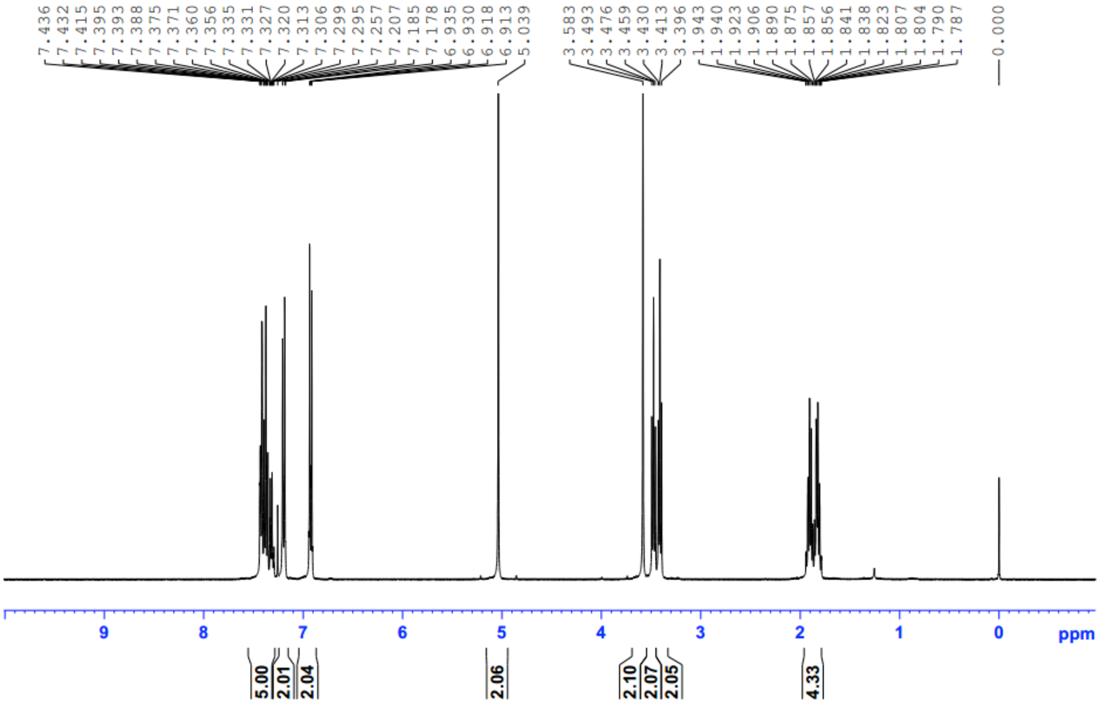

Lyh-E-12-13C
CDCl3
2010.12.01

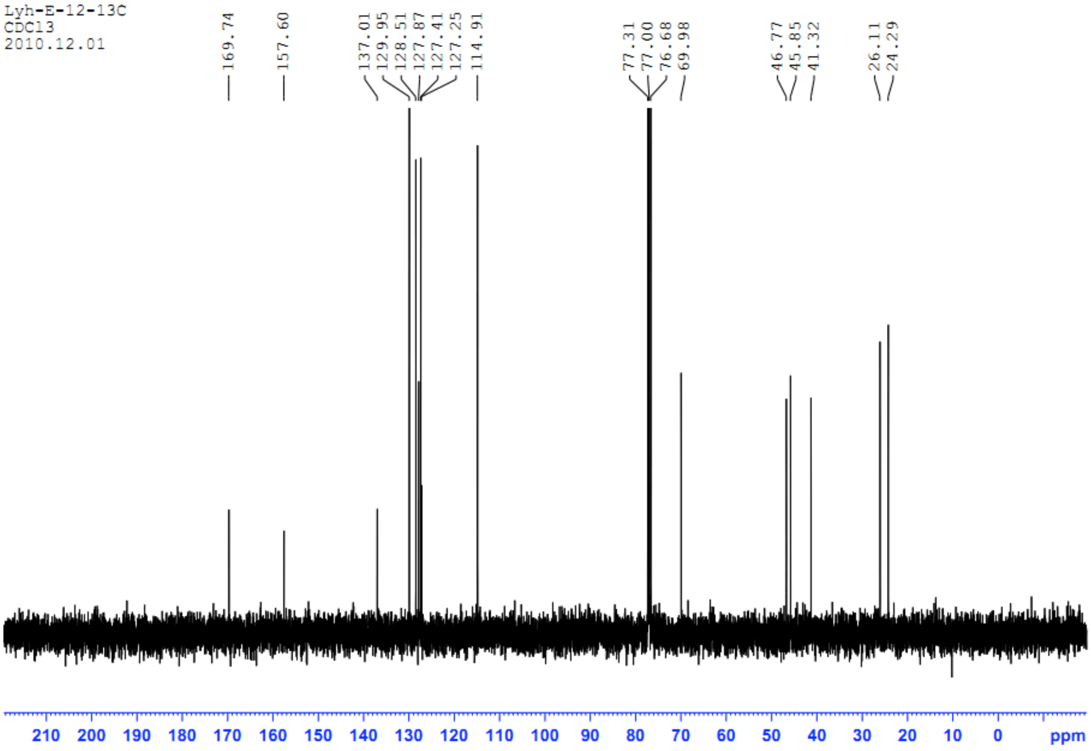

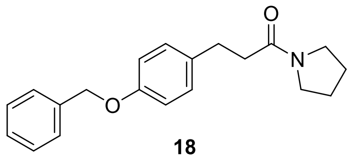

18

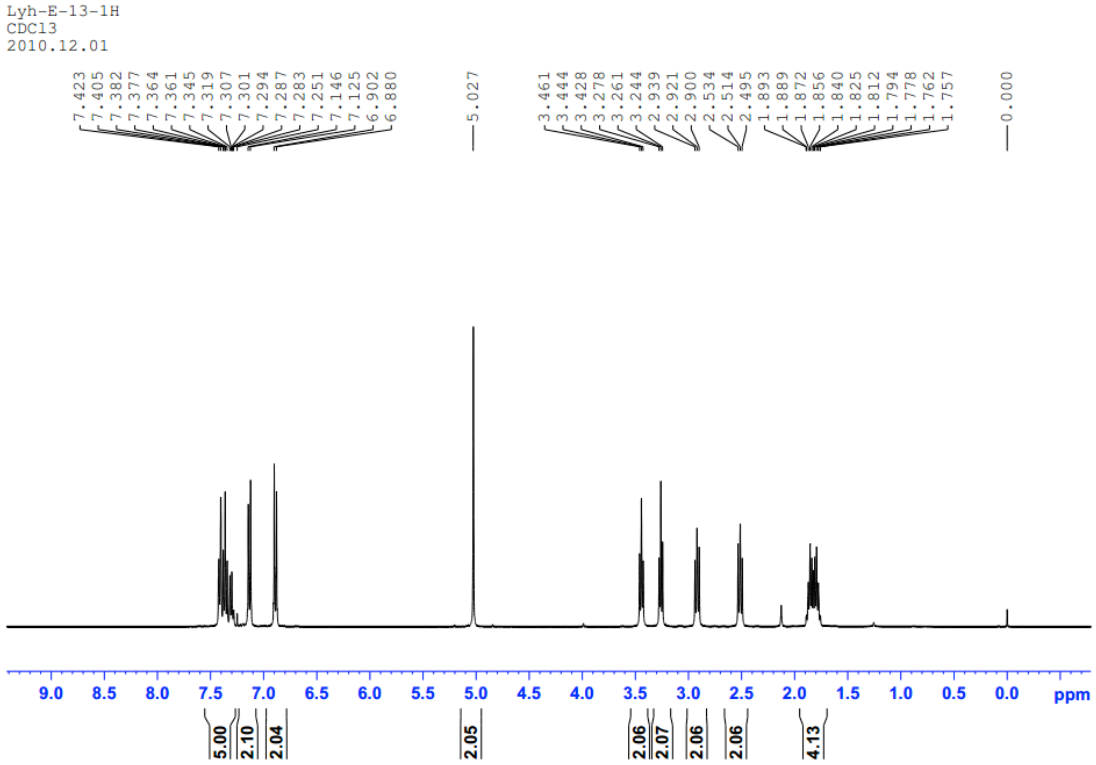
Lyh-E-13-1H
CDCl3
2010.12.01
7.423
7.405
7.382
7.377
7.364
7.361
7.345
7.319
7.307
7.301
7.294
7.287
7.283
7.251
7.146
7.125
6.902
6.880
5.027
3.461
3.444
3.428
3.278
3.261
3.244
2.939
2.921
2.900
2.534
2.514
2.495
1.893
1.889
1.872
1.856
1.840
1.825
1.812
1.794
1.778
1.762
1.757
0.000
9.0 8.5 8.0 7.5 7.0 6.5 6.0 5.5 5.0 4.5 4.0 3.5 3.0 2.5 2.0 1.5 1.0 0.5 0.0 ppm
5.00
2.10
2.04
2.05
2.06
2.07
2.06
2.06
4.13

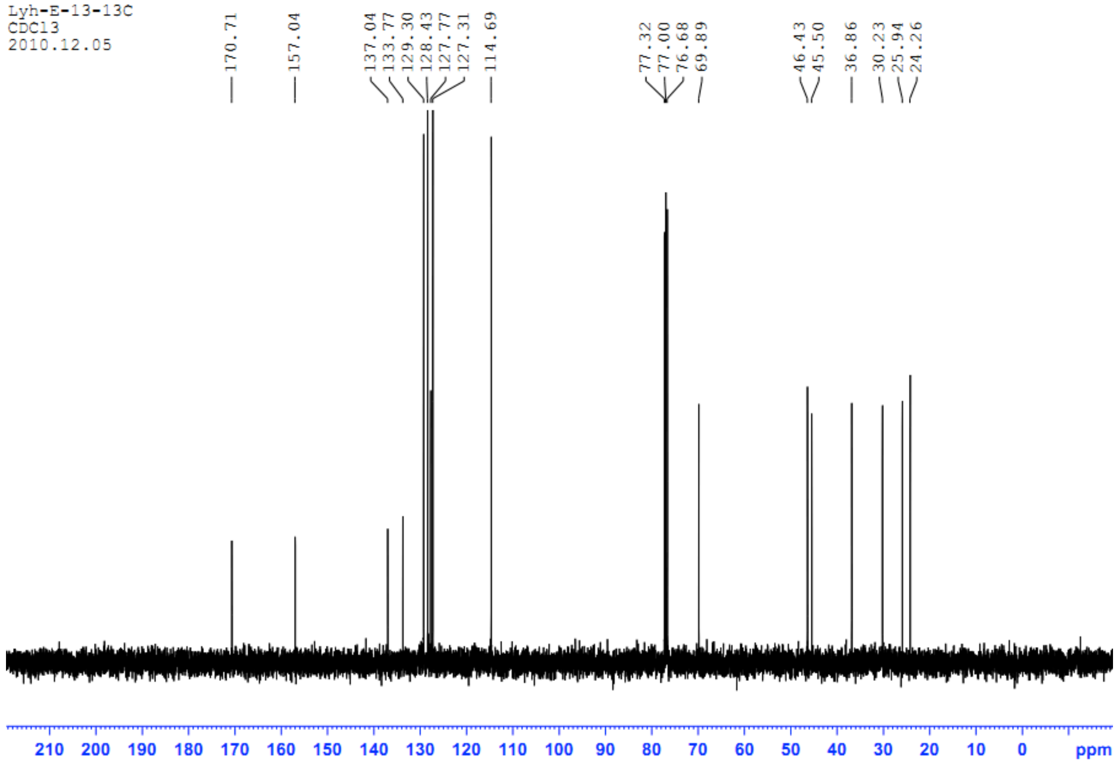
Lyh-E-13-13C
CDCl3
2010.12.05
170.71
157.04
137.04
133.77
129.30
128.43
127.77
127.31
114.69
77.32
77.00
76.68
69.89
46.43
45.50
36.86
30.23
25.94
24.26
210 200 190 180 170 160 150 140 130 120 110 100 90 80 70 60 50 40 30 20 10 0 ppm

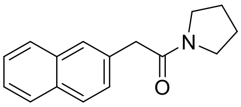

20

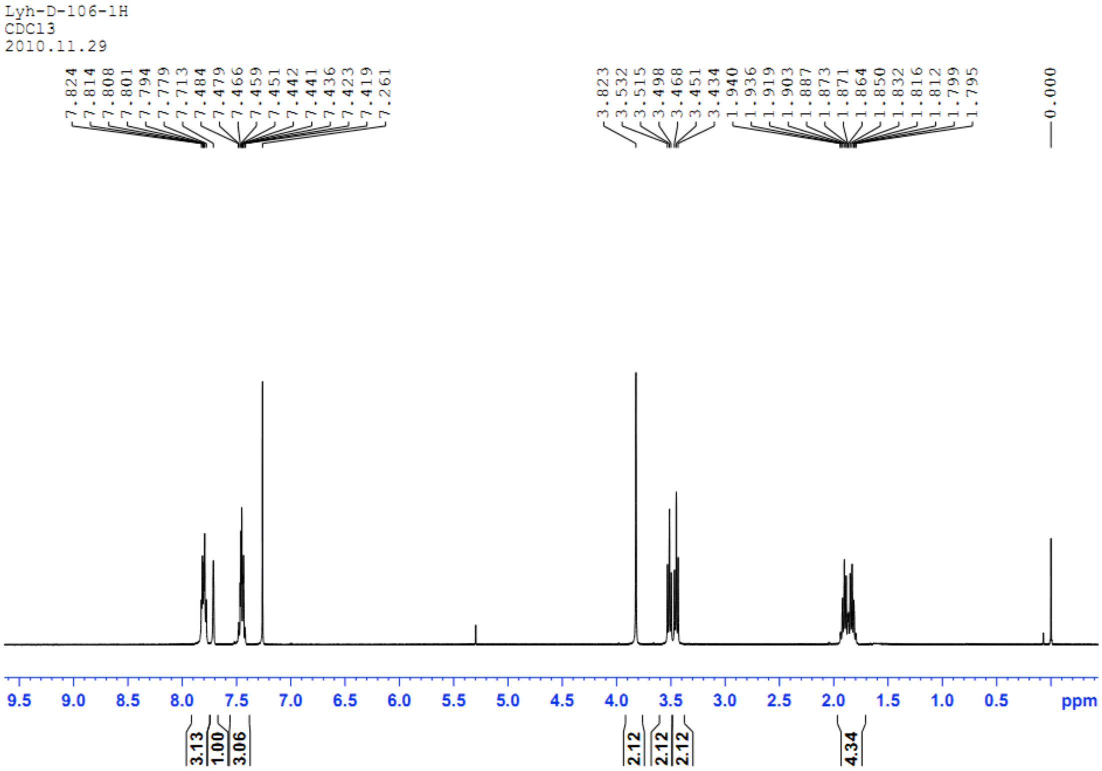
Lyh-D-106-1H
CDCl3
2010.11.29
7.824 7.814 7.808 7.801 7.794 7.779 7.713 7.484 7.479 7.466 7.459 7.451 7.442 7.441 7.436 7.423 7.419 7.261
3.823 3.532 3.515 3.498 3.468 3.451 3.434 1.940 1.936 1.919 1.903 1.887 1.873 1.871 1.864 1.850 1.832 1.816 1.812 1.799 1.795
0.000
9.5 9.0 8.5 8.0 7.5 7.0 6.5 6.0 5.5 5.0 4.5 4.0 3.5 3.0 2.5 2.0 1.5 1.0 0.5 ppm
3.13 1.00 3.06 2.12 2.12 2.12 4.34

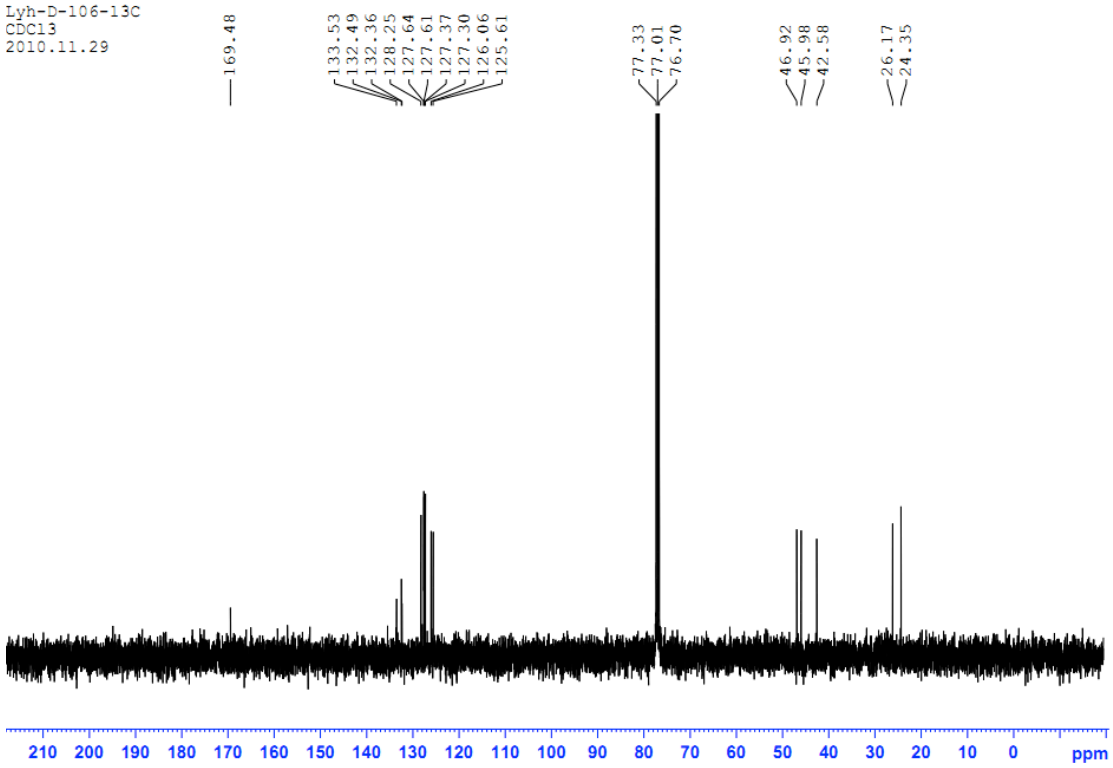
Lyh-D-106-13C
CDCl3
2010.11.29
169.48
133.53 132.49 132.36 128.25 127.64 127.61 127.37 127.30 126.06 125.61
77.33 77.01 76.70
46.92 45.98 42.58
26.17 24.35
210 200 190 180 170 160 150 140 130 120 110 100 90 80 70 60 50 40 30 20 10 0 ppm

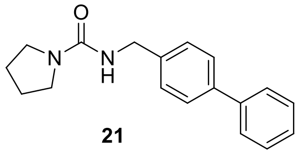

21

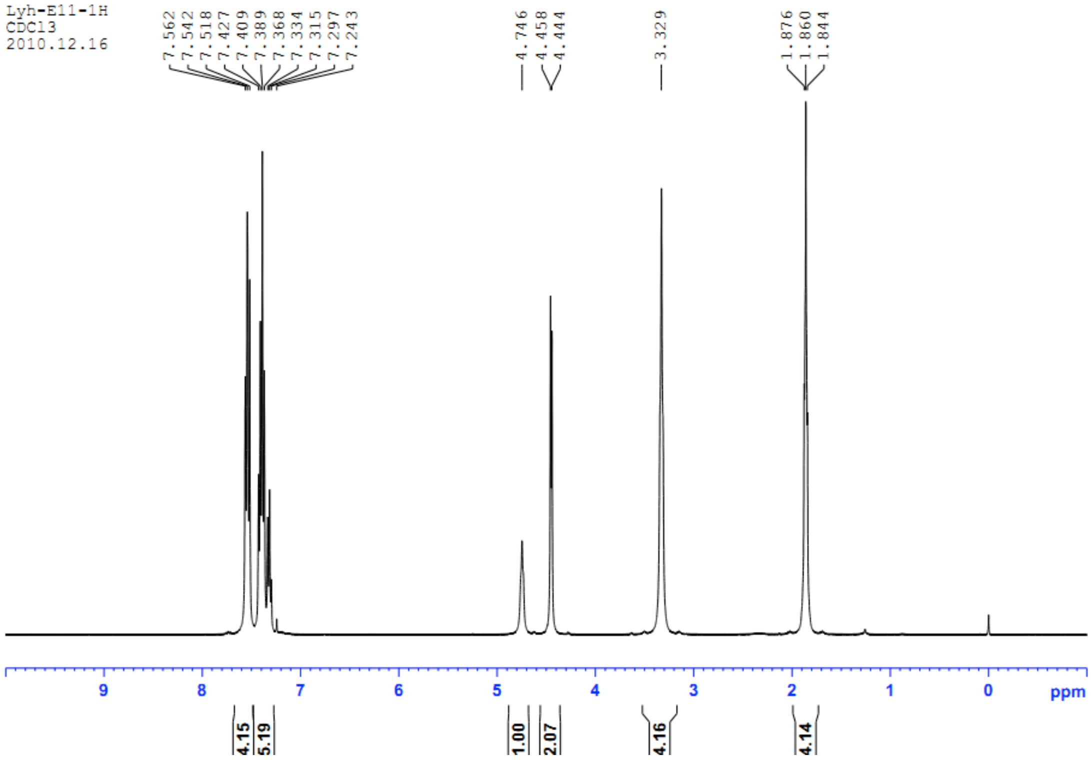
Lyh-E11-1H
CDCl3
2010.12.16
7.562
7.542
7.518
7.427
7.409
7.389
7.368
7.334
7.315
7.297
7.243
4.746
4.458
4.444
3.329
1.876
1.860
1.844
9
8
7
6
5
4
3
2
1
0
ppm
4.15
5.19
1.00
2.07
4.16
4.14

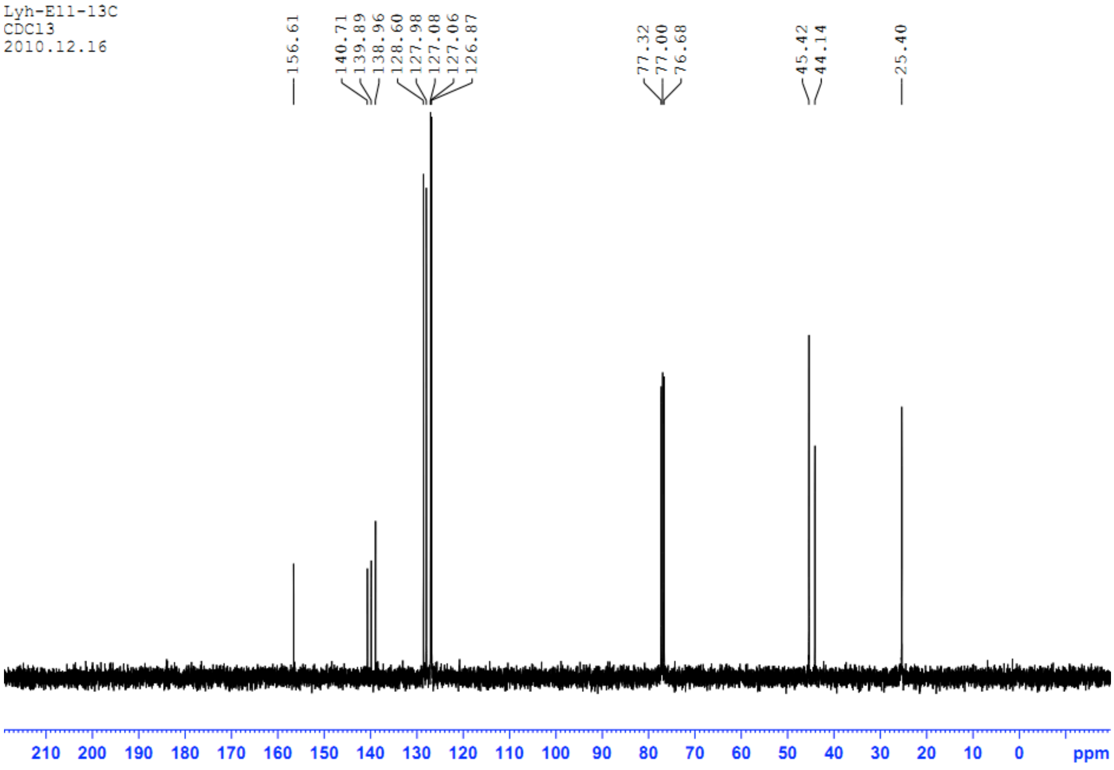
Lyh-E11-13C
CDCl3
2010.12.16
156.61
140.71
139.89
138.96
128.60
127.98
127.08
127.06
126.87
77.32
77.00
76.68
45.42
44.14
25.40
210
200
190
180
170
160
150
140
130
120
110
100
90
80
70
60
50
40
30
20
10
0
ppm

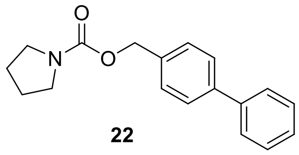

22

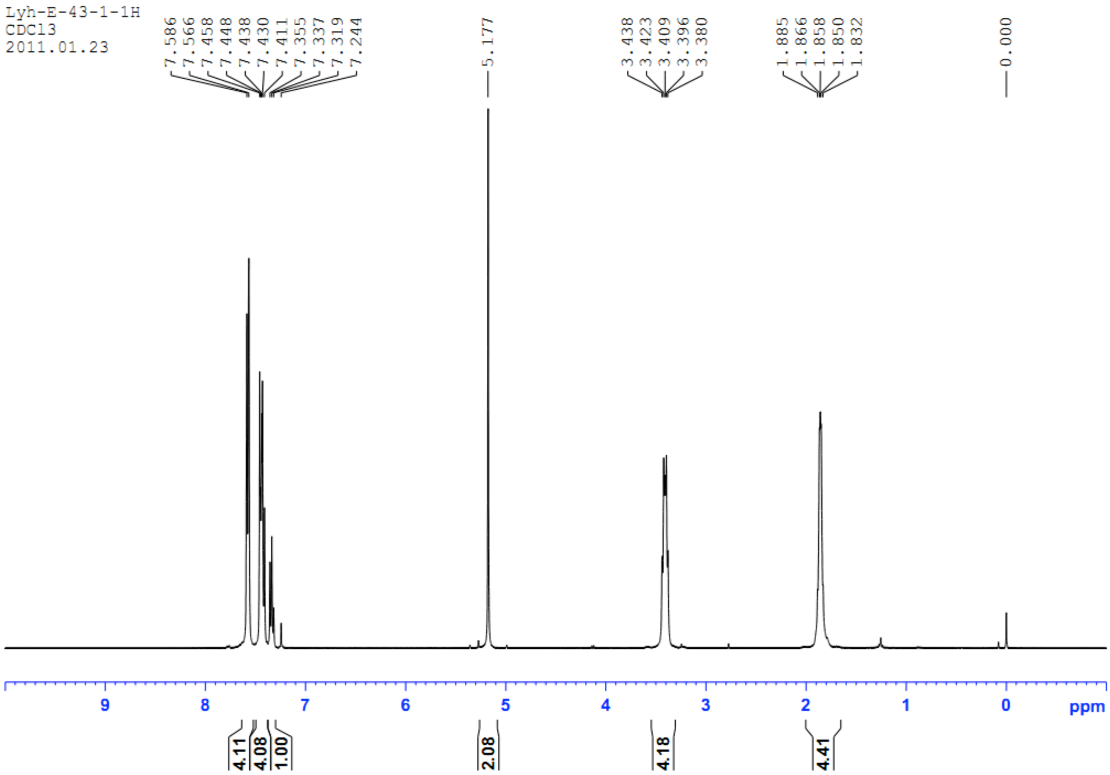

Lyh-E-43-1-1H
CDCl3
2011.01.23
7.586 7.566 7.458 7.448 7.438 7.430 7.411 7.355 7.337 7.319 7.244
5.177
3.438 3.423 3.409 3.396 3.380
1.885 1.866 1.858 1.850 1.832
0.000
9 8 7 6 5 4 3 2 1 0 ppm
4.11 4.08 1.00 2.08 4.18 4.41

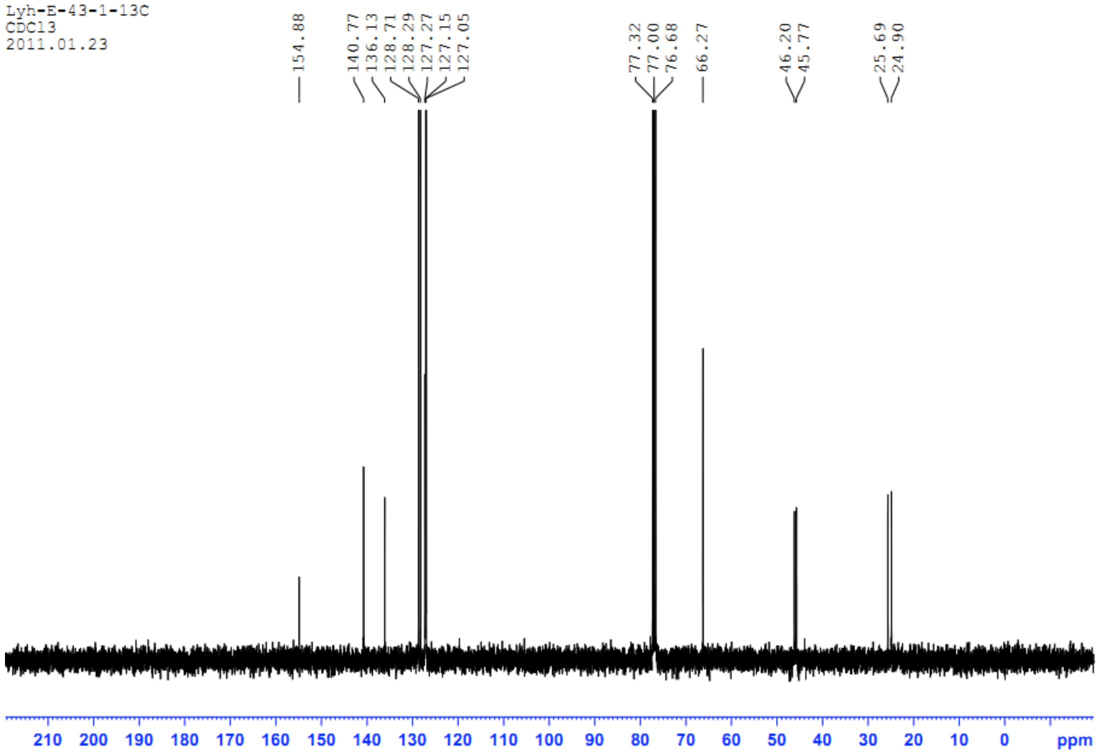

Lyh-E-43-1-13C
CDCl3
2011.01.23
154.88
140.77 136.13 128.71 128.29 127.27 127.15 127.05
77.32 77.00 76.68
66.27
46.20 45.77
25.69 24.90
210 200 190 180 170 160 150 140 130 120 110 100 90 80 70 60 50 40 30 20 10 0 ppm

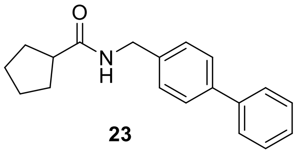

23

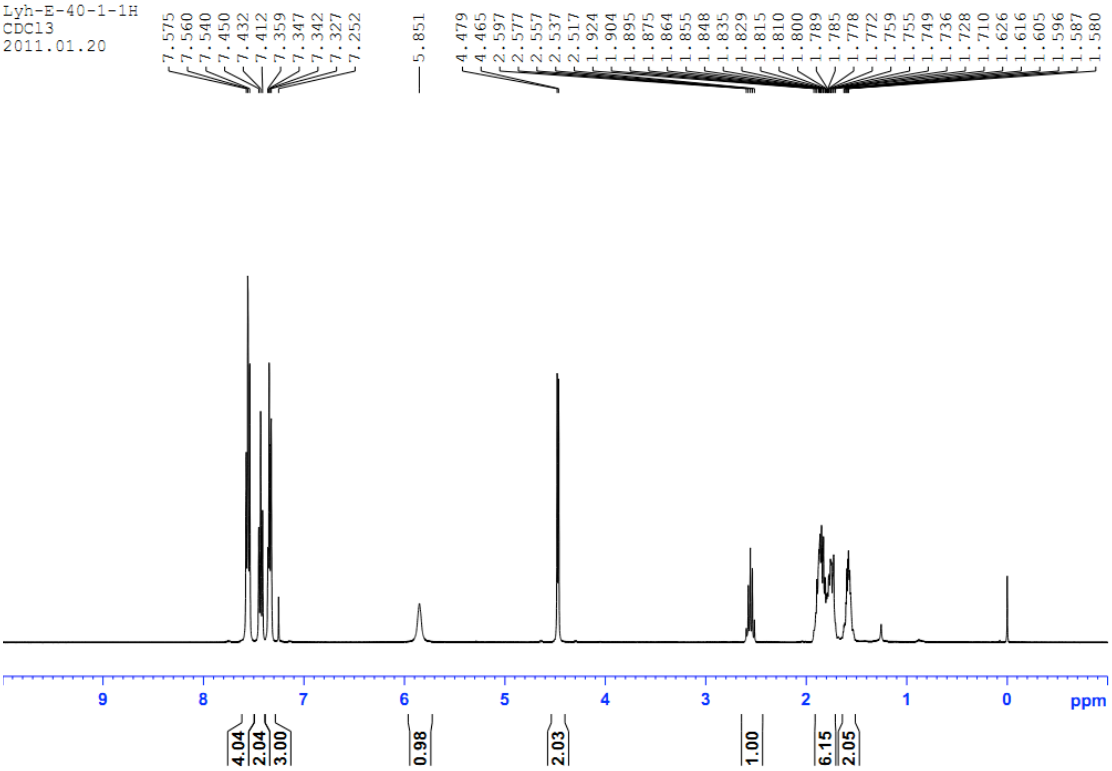
Lyh-E-40-1-1H
CDCl3
2011.01.20
7.575
7.560
7.540
7.450
7.432
7.412
7.359
7.347
7.342
7.327
7.252
5.851
4.479
4.465
2.597
2.577
2.557
2.537
2.517
1.924
1.904
1.895
1.875
1.864
1.855
1.848
1.835
1.829
1.815
1.810
1.800
1.789
1.785
1.778
1.772
1.759
1.755
1.749
1.736
1.728
1.710
1.626
1.605
1.596
1.587
1.580
9
8
7
6
5
4
3
2
1
0
ppm
4.04
2.04
3.00
0.98
2.03
1.00
6.15
2.05

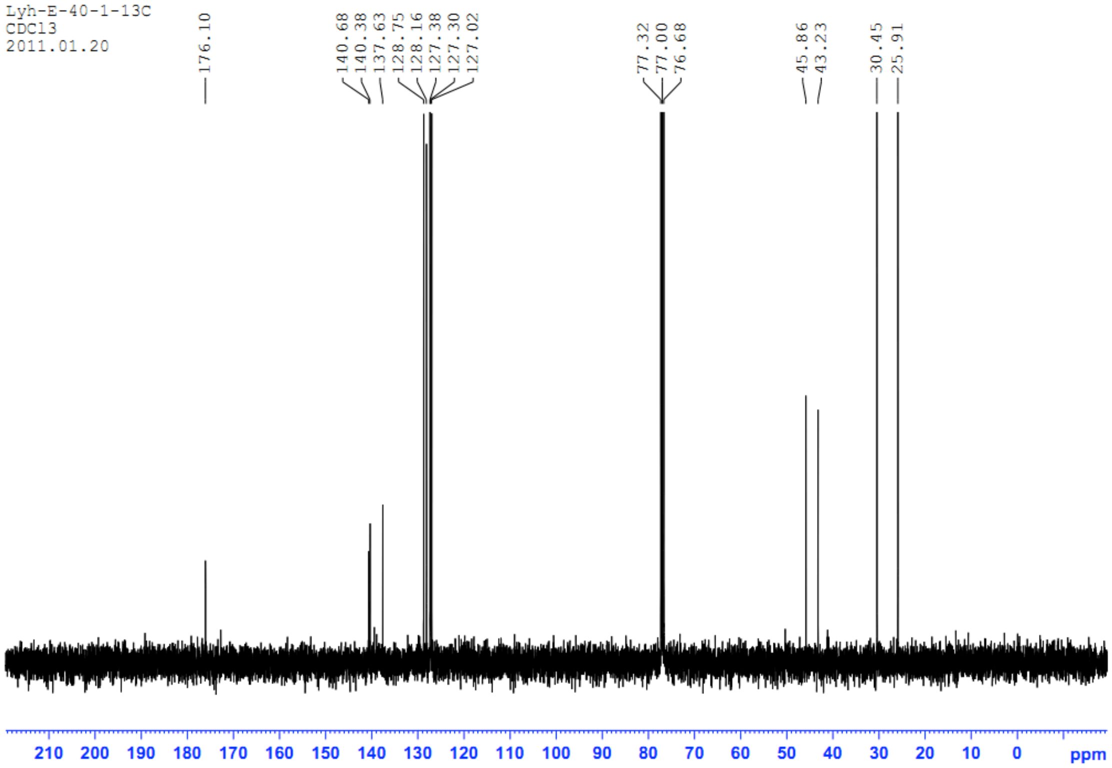
Lyh-E-40-1-13C
CDCl3
2011.01.20
176.10
140.68
140.38
137.63
128.75
128.16
127.38
127.30
127.02
77.32
77.00
76.68
45.86
43.23
30.45
25.91
210
200
190
180
170
160
150
140
130
120
110
100
90
80
70
60
50
40
30
20
10
0
ppm

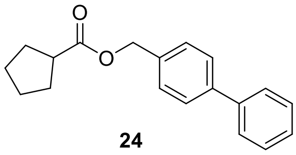

24

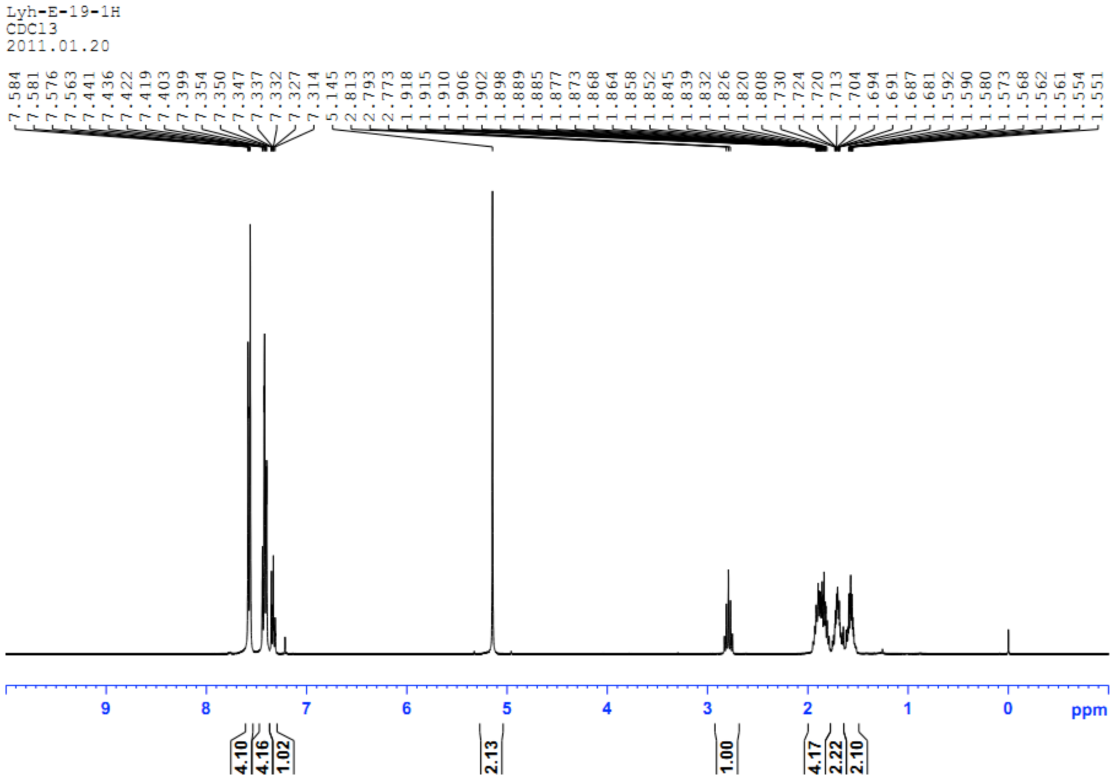
Lyh-E-19-1H
CDCl3
2011.01.20
7.584
7.581
7.576
7.563
7.441
7.436
7.422
7.419
7.403
7.399
7.354
7.350
7.347
7.337
7.332
7.327
7.314
5.145
2.813
2.793
2.773
1.918
1.915
1.910
1.906
1.902
1.898
1.889
1.885
1.877
1.873
1.868
1.864
1.858
1.852
1.845
1.839
1.832
1.826
1.820
1.808
1.730
1.724
1.720
1.713
1.704
1.694
1.691
1.687
1.681
1.592
1.590
1.580
1.573
1.568
1.562
1.561
1.554
1.551
9
8
7
6
5
4
3
2
1
0
ppm
4.10
4.16
1.02
2.13
1.00
4.17
2.22
2.10

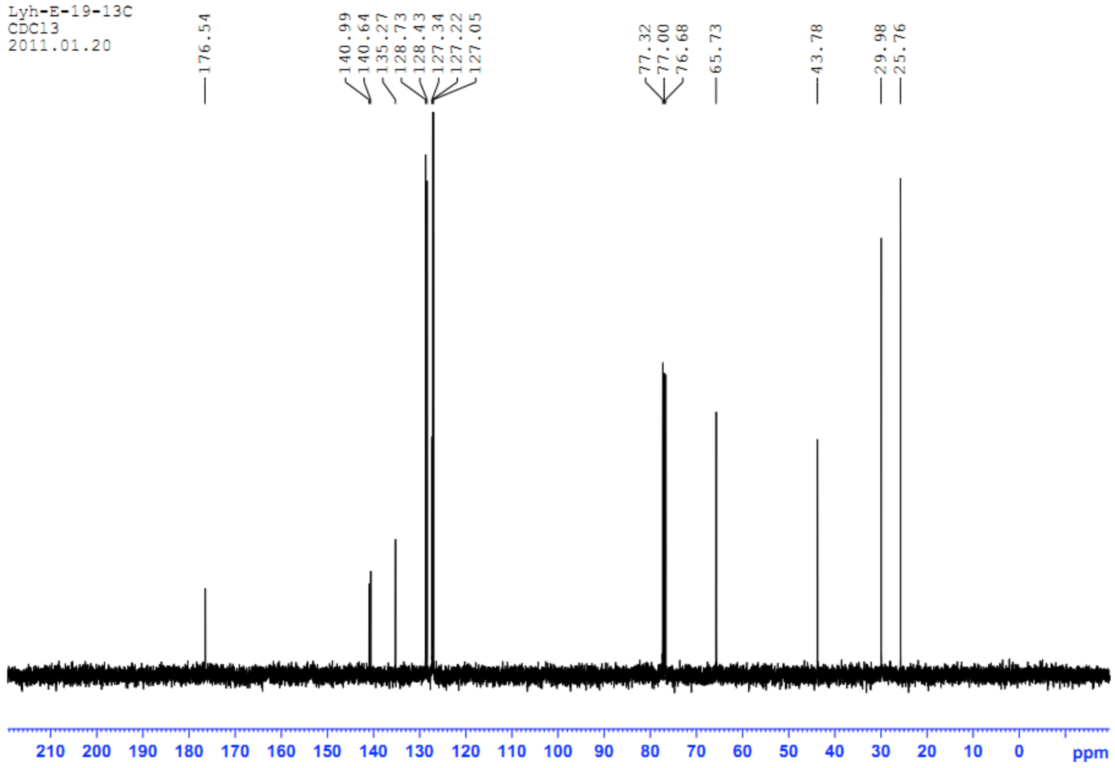
Lyh-E-19-13C
CDCl3
2011.01.20
176.54
140.99
140.64
135.27
128.73
128.43
127.34
127.22
127.05
77.32
77.00
76.68
65.73
43.78
29.98
25.76
210
200
190
180
170
160
150
140
130
120
110
100
90
80
70
60
50
40
30
20
10
0
ppm

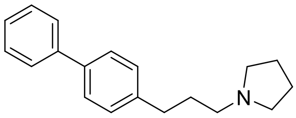

25

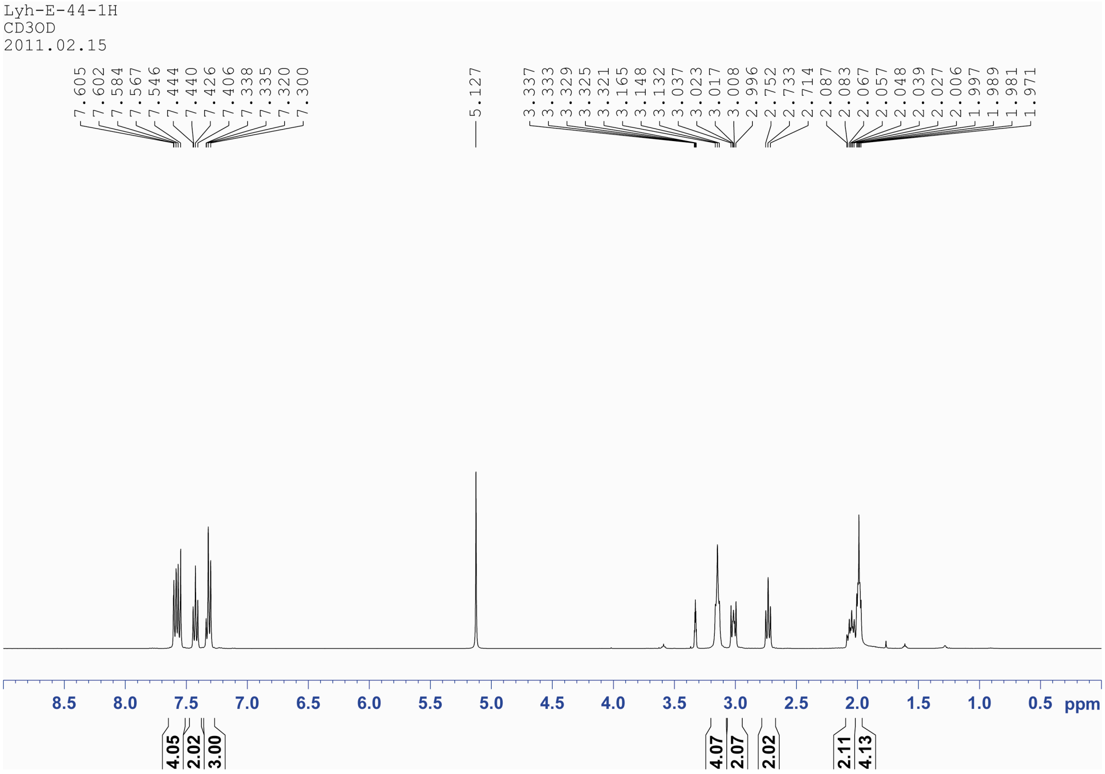
Lyh-E-44-1H
CD3OD
2011.02.15
7.605 7.602 7.584 7.567 7.546 7.444 7.440 7.426 7.406 7.338 7.335 7.320 7.300
5.127
3.337 3.333 3.329 3.325 3.321 3.165 3.148 3.132 3.037 3.023 3.017 3.008 2.996 2.752 2.733 2.714 2.087 2.083 2.067 2.057 2.048 2.039 2.027 2.006 1.997 1.989 1.981 1.971
8.5 8.0 7.5 7.0 6.5 6.0 5.5 5.0 4.5 4.0 3.5 3.0 2.5 2.0 1.5 1.0 0.5 ppm
4.05 2.02 3.00 4.07 2.07 2.02 2.11 4.13

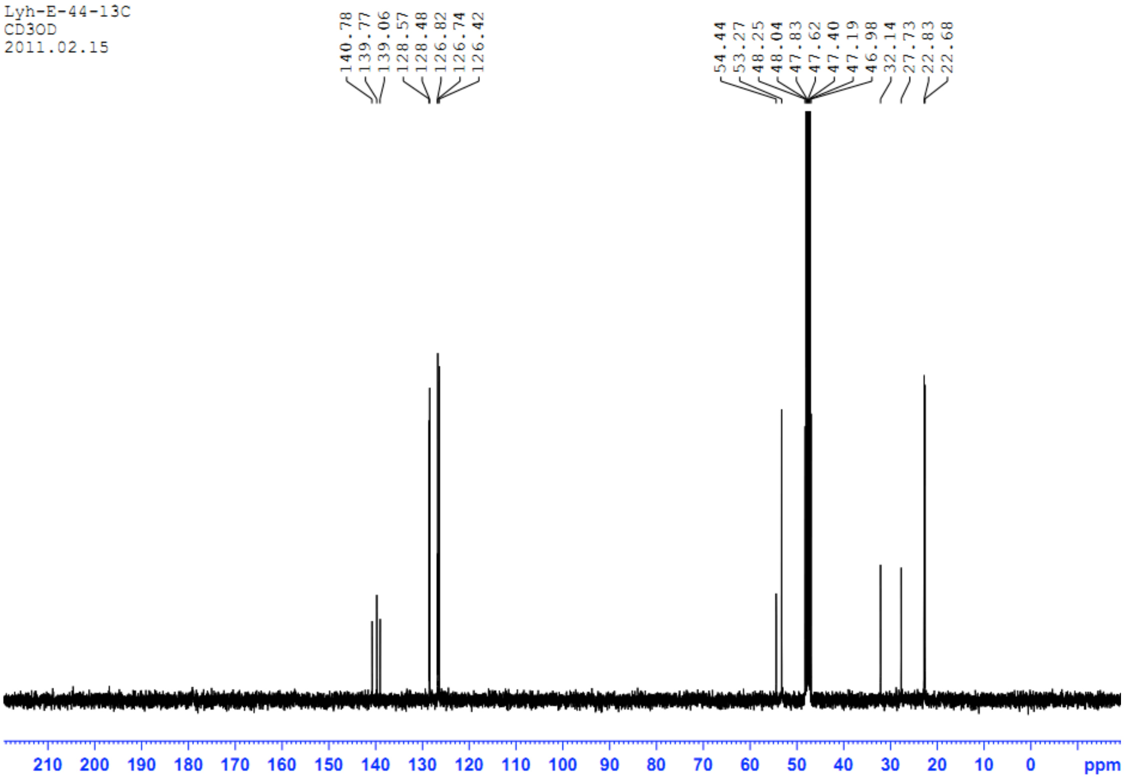
Lyh-E-44-13C
CD3OD
2011.02.15
140.78 139.77 139.06 128.57 128.48 126.82 126.74 126.42
54.44 53.27 48.25 48.04 47.83 47.62 47.40 47.19 46.98 32.14 27.73 22.83 22.68
210 200 190 180 170 160 150 140 130 120 110 100 90 80 70 60 50 40 30 20 10 0 ppm
