# Supplementary material for: Design and Synthesis of Potent N-Acylethanolamine-hydrolyzing Acid Amidase (NAAA) Inhibitor as Anti-Inflammatory Compounds
Source: PLoS One. 2012 Aug 20;7(8):e43023. doi: 10.1371/journal.pone.0043023 (PMC3423427; doi:10.1371/journal.pone.0043023)
Supplement: Table S1 — Inhibition of compounds (1)–(6) on NAAA and FAAH activities. (DOC) [file pone.0043023.s004.doc]

| **Table S1.** Inhibition of compounds (1)–(6) on NAAA and FAAH activities | | | |
| --- | --- | --- | --- |
|  | | | |
| **Compounds** | **R_1_** | **IC_50_ of NAAA (μM)** | **IC_50_ of FAAH (μM)** |
| 1 |  | 25.01 ± 5.7 | 21.78 ± 4.45 |
| 2 |  | >100 | >100 |
| 3 |  | >100 | >100 |
| 4 |  | >100 | >100 |
| 5 |  | >100 | >100 |
| 6 |  | >100 | >100 |
| Data present as IC_50_ ± S.E.M. All experiments were performed triplicate. | | | |
