# Supplementary material for: Design and Synthesis of Potent N-Acylethanolamine-hydrolyzing Acid Amidase (NAAA) Inhibitor as Anti-Inflammatory Compounds
Source: PLoS One. 2012 Aug 20;7(8):e43023. doi: 10.1371/journal.pone.0043023 (PMC3423427; doi:10.1371/journal.pone.0043023)
Supplement: Table S2 — Inhibition of compounds (7)–(20) on NAAA and FAAH activities. (DOC) [file pone.0043023.s005.doc]

| **Table S2.** Inhibition of compounds (7)–(20) on NAAA and FAAH activities | | | |
| --- | --- | --- | --- |
|  | | | |
| **Compounds** | **R_2_** | **IC_50_ of NAAA (μM)** | **IC_50_ of FAAH (μM)** |
| 7 |  | >100 | >100 |
| 8 |  | >100 | >100 |
| 9 |  | >100 | >100 |
| 10 |  | 38.20 ± 3.79 | >100 |
| 11 |  | 34.58 ± 4.82 | >100 |
| 12 |  | 12.92 ± 2.47 | >100 |
| 13 |  | 37.93 ± 4.58 | >100 |
| 14 |  | >100 | >100 |
| 15 |  | >100 | >100 |
| 16 |  | 2.12 ± 0.41 | >100 |
| 17 |  | >100 | >100 |
| 18 |  | >100 | >100 |
| 19 |  | >100 | >100 |
| 20 |  | >100 | >100 |
| Data present as IC_50_ ± S.E.M. All experiments were performed triplicate. | | | |
