# Supplementary material for: Design and Synthesis of Potent N-Acylethanolamine-hydrolyzing Acid Amidase (NAAA) Inhibitor as Anti-Inflammatory Compounds
Source: PLoS One. 2012 Aug 20;7(8):e43023. doi: 10.1371/journal.pone.0043023 (PMC3423427; doi:10.1371/journal.pone.0043023)
Supplement: Table S3 — Inhibition of compounds (21)–(25) on NAAA and FAAH activities. (DOC) [file pone.0043023.s006.doc]

| **Table S3.** Inhibition of compounds (21)–(25) on NAAA and FAAH activities | | | | | |
| --- | --- | --- | --- | --- | --- |
|  | | | | | |
| **Compounds** | **X** | **Y** | **Z** | **IC_50_ of NAAA (μM)** | **IC_50_ of FAAH (μM)** |
| 16 | CH_2_ | O | N | 2.12 ± 0.41 | >100 |
| 21 | NH | O | N | >100 | >100 |
| 22 | O | O | N | >100 | >100 |
| 23 | NH | O | CH_2_ | >100 | >100 |
| 24 | O | O | CH_2_ | >100 | >100 |
| 25 | CH_2_ | H | N | >100 | >100 |
| Data present as IC_50_ ± S.E.M. All experiments were performed triplicate. | | | | | |
