# Supplementary material for: Design and Synthesis of Potent N-Acylethanolamine-hydrolyzing Acid Amidase (NAAA) Inhibitor as Anti-Inflammatory Compounds
Source: PLoS One. 2012 Aug 20;7(8):e43023. doi: 10.1371/journal.pone.0043023 (PMC3423427; doi:10.1371/journal.pone.0043023)
Supplement: Table S4 — The stability of compound 16. (DOC) [file pone.0043023.s007.doc]

| **Table S4.** The stability of compound 16 | | | | | |
| --- | --- | --- | --- | --- | --- |
|  | 0.1M HCl | 0.1NaOH | 80°C | Rat Serum | |
| Compound 16 | 100% | 100% | 100% | 8h | 16h |
|  |  |  |  | 89% | 64% |
