# Supplementary material for: Design and Synthesis of Potent N-Acylethanolamine-hydrolyzing Acid Amidase (NAAA) Inhibitor as Anti-Inflammatory Compounds
Source: PLoS One. 2012 Aug 20;7(8):e43023. doi: 10.1371/journal.pone.0043023 (PMC3423427; doi:10.1371/journal.pone.0043023)
Supplement: Text S1 — Supplementary Methods and Supplementary References. (DOC) [file pone.0043023.s008.doc]

**Supplementary Methods**

**1–Pentadecanyl–carbonyl pyrrolidine (1) ^(1)^**

Following the general **method A** (eluent: EtOAc/PE 1: 5), the amidation of palmitic acid with pyrrolidine afforded **1** (79 mg; yield: 89%) as white crystals: Mp 84.0–84.6°C; IR (film) *ν*_max_: 2923, 2852, 1648, 1426 cm^–1^; ^1^H NMR (400 MHz, CDCl_3_) δ 0.88 (t, *J* = 7.2 Hz, 3 H), 1.25–1.30 (m, 24 H), 1.60–1.67 (m, 2 H), 1.81–1.88 (m, 2 H), 1.91–1.98 (m, 2 H), 2.25 (t, *J* = 8 Hz, 2 H), 3.39–3.47 (m, 4 H) ppm; ^13^C NMR (100 MHz, CDCl_3_) δ 14.1, 22.6, 24.4, 24.9, 26.1, 29.3, 29.43, 29.49, 29.52, 29.62, 29.64, 31.9, 34.8, 45.5, 46.6, 171.8 ppm; MS (ESI, *m/z*): 310 (M + H^+^).

***N*–Cyclopentylpalmitamide (2)**

Following the general **method A** (eluent: EtOAc/PE 1: 3), the amidation of palmitic acid with cyclopentanamine afforded **2** (82 mg; yield: 85%) as white crystals: Mp 62.4–63.6°C; IR (film) *ν*_max_: 3304, 2918, 2850, 1639, 1544, 1471 cm^–1^; ^1^H NMR (400 MHz, CDCl_3_) δ 0.88 (t, *J* = 6.8 Hz, 3 H), 1.21–1.48 (m, 26 H), 1.56–1.70 (m, 6 H), 1.95–2.03 (m, 2 H), 2.12 (t, *J* = 8.0 Hz, 2 H), 4.16–4.25 (m, 1 H), 5.40 (br, 1 H) ppm; ^13^C NMR (100 MHz, CDCl_3_) δ 14.1, 22.7, 23.7, 25.8, 29.28, 29.33, 29.5, 29.6, 29.62, 29.66, 31.9, 33.2, 37.0, 51.0, 172.7 ppm; MS (ESI, *m/z*): 324 (M + H^+^); Anal. calcd for C_21_H_41_NO: C, 77.95; H, 12.77; N, 4.33. Found: C, 77.79; H, 12.74; N, 4.34.

1. **Pentadecanyl–carbonyl piperidine (3)**

Following the general **method A** (eluent: EtOAc/PE 1: 5), the amidation of palmitic acid with piperidine afforded **3** (84 mg; yield: 87%) as white crystals: Mp 36.5–37.1°C; IR (film) *ν*_max_: 2924, 2853, 1649, 1432, 1253, 1220 cm^–1^; ^1^H NMR (400 MHz, CDCl_3_) δ 0.88 (t, *J* = 6.4 Hz, 3 H), 1.25–1.30 (m, 24 H), 1.52–1.65 (m, 8 H), 2.30 (t, *J* = 8.0 Hz, 2 H), 3.39 (t, *J* = 7.2 Hz, 2 H), 3.54 (t, *J* = 7.2 Hz, 2 H) ppm; ^13^C NMR (100 MHz, CDCl_3_) δ 14.0, 22.6, 24.5, 25.4, 25.5, 26.5, 29.2, 29.3, 29.41, 29.44, 29.51, 29.54, 29.6, 31.8, 33.4, 42.5, 46.6, 171.4 ppm; MS (ESI, *m/z*): 324(M + H^+^); Anal. calcd for C_21_H_41_NO: C, 77.95; H, 12.77; N, 4.33. Found: C, 77.84; H, 12.80; N, 4.33.

**Cyclopentyl palmitate (4)**

Following the general **method A** (eluent: EtOAc/PE 1: 30), the esterification of palmitic acid with cyclopentanol afforded **4** (88 mg; yield: 91%) as colorless oil; IR (film) *ν*_max_: 2924, 2853, 1735, 1466, 1161 cm^–1^; ^1^H NMR (400 MHz, CDCl_3_) δ 0.88 (t, *J* = 6.8 Hz, 3 H), 1.26–1.28 (m, 26 H), 1.56–1.76 (m, 6 H), 1.81–1.89 (m, 2 H), 2.26 (t, *J* = 7.2 Hz, 2 H), 5.13–5.18 (m, 1 H) ppm; ^13^C NMR (100 MHz, CDCl_3_) δ 14.1, 22.7, 23.7, 25.1, 29.1, 29.3, 29.4, 29.5, 29.59, 29.64, 29.7, 31.9, 32.6, 34.7, 76.7, 173.7 ppm; MS (ESI, *m/z*): 325 (M + H^+^); Anal. calcd for C_21_H_40_O_2_: C, 77.72; H, 12.42. Found: C, 77.95; H, 12.44.

**Tetrahydrofuran–3–yl palmitate (5)**

Following the general **method A** (eluent: EtOAc/PE 1: 30), the esterification of palmitic acid with tetrahydrofuran–3–ol afforded **5** (79 mg; 81%) as white crystals: Mp 43.9–44.3°C; IR (film) *ν*_max_: 2922, 2857, 1733, 1464, 1379, 1179, 1078 cm^–1^; ^1^H NMR (400 MHz, CDCl_3_) δ 0.88 (t, *J* = 6.8 Hz, 3 H), 1.25–1.29 (m, 24 H), 1.58–1.63 (m, 2 H), 1.95–2.02 (m, 1 H), 2.12–2.21 (m, 1 H), 2.29 (t, *J* = 7.2 Hz, 2 H), 3.78–3.94 (m, 4 H), 5.27–5.31 (m, 1 H) ppm; ^13^C NMR (100 MHz, CDCl_3_) δ 14.1, 22.7, 24.9, 29.1, 29.2, 29.3, 29.4, 29.57, 29.63, 29.7, 31.9, 32.8, 34.4, 67.0, 73.2, 74.5, 173.6 ppm; MS (ESI, *m/z*): 327 (M + H^+^); Anal. calcd for C_20_H_38_O_3_: C, 73.57; H, 11.73; Found: C, 73.42; H, 11.77.

**1–Pentadecanyl–carbonyl pyrrole (6)**

To a solution of freshly distilled pyrrole (34 mg, 0.5 mmol) in THF (5 mL) was added slowly a solution of 2.5 M n–BuLi (0.55 mmol, 0.2 mL hexane) under nitrogen atmosphere at –78°C. After being stirred at the same temperature for 10 min, a solution of palmitoyl chloride (151 mg, 0.55 mmol) in THF (1 mL) was slowly added. The reaction mixture was stirred at –78°C for 0.5 hours, and allowed to warm slowly to room temperature in 5 hours. The reaction was quenched with 1.0 mL a sat. aqueous solution of NH_4_Cl and extracted with EtOAc (3 × 5 mL). The combined organic layers were dried over anhydrous Na_2_SO_4_, filtered and concentrated under reduced pressure. The residue was purified by flash chromatography on silica gel (eluent: EtOAc/PE 1: 100) to give compound **6** (139 mg, yield: 92%) as white crystals: Mp: 57.9–60.2°C; IR (film) *ν*_max_: 2915, 2844, 1710, 1460, 740, 709 cm^–1^; ^1^H NMR (400 MHz, CDCl_3_) δ 0.88 (t, *J* = 6.4 Hz, 3 H), 1.26–1.41 (m, 24 H), 1.74–1.81 (m, 2 H), 2.80 (t, *J* = 7.2 Hz, 2 H), 6.28 (t, *J* = 2.4 Hz, 2 H), 7.31 (br, 2 H) ppm; ^13^C NMR (100 MHz, CDCl_3_) δ 14.1, 22.7, 24.6, 29.2, 29.3, 29.4, 29.5, 29.6, 29.67, 29.69, 31.9, 34.6, 112.9, 119.0, 170.6 ppm; MS (ESI, *m/z*): 306 (M + H^+^); Anal. calcd for C_20_H_35_NO: C, 78.63; H, 11.55; N, 4.58; Found: C, 78.85; H, 11.56; N, 4.58.

**1–Benzyl–carbonyl pyrrolidine (7) ^(2)^**

Following the general **method A** (eluent: EtOAc/PE 1:3), the amidation of 2–phenylacetic acid with pyrrolidine afforded **7** (50 mg, 82%) as colorless oil: IR (film) *ν*_max_: 3015, 2972, 2902, 2872, 1635, 1433, 1022 cm^–1^; ^1^H NMR (400 MHz, CDCl_3_) δ 1.76–1.99 (m, 4 H), 3.33–3.51 (m, 6 H), 7.20–7.28 (m, 5 H) ppm; ^13^C NMR (100 MHz, CDCl_3_) δ 23.9, 25.8, 41.5, 46.2, 46.4, 125.9, 128.1, 128.2, 139.4, 169.1 ppm; MS (ESI, *m/z*): 190 (M +H^+^).

**1–(2–Phenylethyl)–carbonyl pyrrolidine (8) ^(3)^**

Following the general **method A** (eluent: EtOAc/PE 1:4), the amidation of 3–phenylpropanoic acid with pyrrolidine afforded **8** (48 mg, 79%) as colorless oil: IR (film) *ν*_max_: 3007, 2921, 2872, 1637, 1426, 1017 cm^–1^; ^1^H NMR (400 MHz, CDCl_3_) δ 1.77–1.91 (m, 4 H), 2.55 (t, *J* = 8.0 Hz, 2 H), 2.98 (t, *J* = 8.0 Hz, 2 H), 3.27 (t, *J* = 6.8 Hz, 2 H), 3.45 (t, *J* = 6.8 Hz, 2 H), 7.16–7.29 (m, 5 H) ppm; ^13^C NMR (100 MHz, CDCl_3_) δ 24.2, 25.8, 31.0, 36.6, 45.5, 46.4, 125.9, 128.2, 128.3, 141.4, 170.6 ppm; MS (ESI, *m/z*): 204 (M + H^+^).

**1–(3–Phenylpropanyl)–carbonyl pyrrolidine (9) ^(4)^**

Following the general **method A** (eluent: EtOAc/PE 1:4), the amidation of 4–phenylbutanoic acid with pyrrolidine afforded **9** (53 mg, 82%) as colorless oil: IR (film) *ν*_max_: 3008, 2962, 2943, 2859, 1636, 1434, 1028, 752 cm^–1^; ^1^H NMR (400 MHz, CDCl_3_) δ 1.79–2.03 (m, 6 H), 2.26 (t, *J* = 7.2 Hz, 2 H), 2.68 (t, *J* = 7.2 Hz, 2 H), 3.32 (t, *J* = 6.8 Hz, 2 H), 3.45 (t, *J* = 6.8 Hz, 2 H), 7.17–7.20 (m, 3 H), 7.25–7.29 (m, 2 H) ppm; ^13^C NMR (100 MHz, CDCl_3_) δ 24.3, 26.0, 26.1, 33.7, 35.3, 45.5, 46.4, 125.7, 128.2, 128.4, 141.8, 171.2 ppm.

**1–(4–Phenylbutanyl)–carbonyl pyrrolidine (10)**

Following the general **method A** (eluent: EtOAc/PE 1:5), the amidation of 5–phenylpentanoic acid with pyrrolidine afforded **10** (58 mg, 84%) as colorless oil: IR (film) *ν*_max_: 3011, 2937, 2863, 1646, 1431, 742, 695 cm^–1^; ^1^H NMR (400 MHz, CDCl_3_) δ 1.64–1.79 (m, 4 H), 1.83 (tt, *J* = 6.4, 6.8 Hz, 2 H), 1.88 (tt, *J* = 6.4, 6.8 Hz, 2 H), 2.27 (t, *J* = 7.2 Hz, 2 H), 2.64 (t, *J* = 7.2 Hz, 2 H), 3.37 (t, *J* = 6.8 Hz, 2 H), 3.45 (t, *J* = 6.8 Hz, 2 H), 7.14–7.28 (m, 5 H) ppm; ^13^C NMR (100 MHz, CDCl_3_) δ 24.3, 24.5, 26.0, 31.2, 34.5, 35.7, 45.5, 46.5, 125.6, 128.1, 128.3, 142.3, 171.4 ppm; MS (ESI, *m/z*): 232 (M + H^+^); HRMS (ESI) calcd for [C_15_H_22_NO]^+^ (M + H^+^): 232.1696; found: 232.1703.

**1–(5–Phenylpentanyl)–carbonyl pyrrolidine (11)**

Following the general **method A** (eluent: EtOAc/PE 1:5), the amidation of 6–phenylhexanoic acid with pyrrolidine afforded **11** (56 mg, 76%) as colorless oil: IR (film) *ν*_max_: 3003, 2932, 2856, 1643, 1432, 752, 700 cm^–1^; ^1^H NMR (400 MHz, CDCl_3_) δ 1.35–1.42 (m, 2 H), 1.61–1.72 (m, 4 H), 1.83 (tt, *J* = 6.0, 7.0 Hz, 2 H), 1.94 (tt, *J* = 6.0, 7.0 Hz, 2 H), 2.24 (t, *J* = 8.0 Hz, 2 H), 2.61 (t, *J* = 8.0 Hz, 2 H), 3.37 (t, *J* = 7.0 Hz, 2 H), 3.45 (t, *J* = 7.0 Hz, 2 H), 7.14–7.18 (m, 3 H), 7.26 (t, *J* = 7.6 Hz, 2 H) ppm; ^13^C NMR (100 MHz, CDCl_3_) δ 24.3, 24.7, 26.0, 29.0, 31.2, 34.6, 35.7, 45.5, 46.5, 125.5, 128.1, 128.3, 142.6, 171.6 ppm; MS (ESI, *m/z*): 246 (M + H^+^); Anal. calcd for C_16_H_23_NO: C, 78.32; H, 9.45; N, 5.71. Found: C, 78.16; H, 9.48; N, 5.70.

**1–(6–Phenylhexanyl)–carbonyl pyrrolidine (12)**

Following the general **method A** (eluent: EtOAc/PE 1:5), the amidation of 7–phenylheptanoic acid with pyrrolidine afforded **12** (69 mg, 89%) as colorless oil; IR (film) *ν*_max_: 3003, 2929, 2850, 1643, 1452, 1429, 755, 695 cm^–1^; ^1^H NMR (400 MHz, CDCl_3_) δ 1.36–1.40 (m, 4 H), 1.58–1.68 (m, 4 H), 1.83 (tt, *J* = 6.4, 7.0 Hz, 2 H), 1.93 (tt, *J* = 6.4, 7.0 Hz, 2 H), 2.23 (t, *J* = 7.2 Hz, 2 H), 2.60 (t, *J* = 7.2 Hz, 2 H), 3.38 (t, *J* = 7.0 Hz, 2 H), 3.45 (t, *J* = 7.0 Hz, 2 H), 7.14–7.17 (m, 3 H), 7.26 (t, *J* = 8.0 Hz, 2 H) ppm; ^13^C NMR (100 MHz, CDCl_3_) δ 24.3, 24.7, 26.0, 29.0, 29.2, 31.2, 34.7, 35.8, 45.5, 46.5, 125.5, 128.1, 128.3, 142.7, 171.6 ppm; MS (ESI, *m/z*): 260 (M + H^+^); Anal. calcd for C_17_H_25_NO: C, 78.72; H, 9.71; N, 5.40. Found: C, 78.52; H, 9.73; N, 5.39.

**1–(7–Phenylheptanyl)–carbonyl pyrrolidine (13)**

Following the general **method A** (eluent: EtOAc/PE 1:5), the amidation of 8–phenyloctanoic acid with pyrrolidine afforded **13** (72 mg, 88%) as colorless oil; IR (film) *ν*_max_: 3011, 2927, 2853, 1643, 1428, 1034, 701 cm^–1^; ^1^H NMR (400 MHz, CDCl_3_) δ 1.36–1.40 (m, 6 H), 1.58–1.67 (m, 4 H), 1.83 (tt, *J* = 6.8, 7.0 Hz, 2 H), 1.93 (tt, *J* = 6.8, 7.0 Hz, 2 H), 2.23 (t, *J* = 8.0 Hz, 2 H), 2.57 (t, *J* = 8.0 Hz, 2 H), 3.38 (t, *J* = 7.0 Hz, 2 H), 3.45 (t, *J* = 7.0 Hz, 2 H), 7.16 (m, 3 H), 7.26 (t, *J* = 6.8 Hz, 2 H) ppm; ^13^C NMR (100 MHz, CDCl_3_) δ 24.3, 24.8, 26.0, 29.1, 29.2, 29.3, 31.4, 34.7, 35.8, 45.5, 46.5, 125.4, 128.1, 128.3, 142.7, 171.7 ppm; MS (ESI, *m/z*): 274 (M + H^+^); Anal. calcd for C_18_H_27_NO: C, 79.07; H, 9.95; N, 5.12. Found: C, 79.34; H, 9.97; N, 5.10.

**1–(Biphenyl–4–ylcarbonyl)pyrrolidine (14) ^(5)^**

Following the general **method A** (eluent: EtOAc/PE 1:3), the amidation of 4–phenylbenzoic acid with pyrrolidine afforded **14** (59 mg, 78%) as white crystals: Mp 124.5–126.9°C; IR (film) *ν*_max_: 3053, 2949, 2869, 1628, 1430, 998, 841, 784 cm^–1^; ^1^H NMR (400 MHz, CDCl_3_) δ 1.85–1.99 (2m, 4 H), 3.47 (t, *J* = 6.4 Hz, 2 H), 3.66 (t, *J* = 6.4 Hz, 2 H), 7.33 (m, 1 H), 7.36 (t, *J* = 7.6 Hz, 2 H), 7.58–7.63 (m, 6 H) ppm; ^13^C NMR (100 MHz, CDCl_3_) δ 24.3, 26.3, 46.1, 49.5, 126.8, 127.0, 127.1, 127.6, 128.7, 135.8, 140.2, 142.5, 169.3 ppm; MS (ESI, *m/z*): 252 (M + H^+^).

**1–(4–Phenylbenzyl)–carbonyl pyrrolidine (15)**

Following the general **method A** (eluent: EtOAc/PE 1:3), the amidation of 2–(biphenyl–4–yl)acetic acid with pyrrolidine afforded **15** (68 mg, 86%) as white crystals: Mp: 115.8–116.9°C, IR (film) *ν*_max_: 3013, 2975, 2869, 1630, 1441, 1374, 1012, 756, 688 cm^–1^; ^1^H NMR (400 MHz, CDCl_3_) δ 1.81–1.96 (m, 4 H), 3.40 (t, *J* = 6.8 Hz, 2 H), 3.50 (t, *J* = 6.8 Hz, 2 H), 3.69 (s, 2 H), 7.25–7.40 (m, 3 H), 7.40–7.46 (m, 2 H), 7.53–7.59 (m, 4 H) ppm; ^13^C NMR (100 MHz, CDCl_3_) δ 24.3, 26.1, 41.8, 46.0, 46.9, 127.0, 127.1, 127.3, 128.7, 129.4, 133.9, 139.6, 140.8, 169.5 ppm; MS (ESI, *m/z*): 266 (M + H^+^); HRMS (ESI) calcd for [C_18_H_20_NO]^+^ (M + H^+^): 266.1539; found: 266.1547.

**1–(2–Biphenyl–4–yl)ethyl–carbonyl pyrrolidine (16)**

Following the general **method A** (eluent: EtOAc/PE 1:2), the amidation of 2–(biphenyl–4–yl)propanoic acid with pyrrolidine afforded **16** (77 mg, 92%) as white crystals: Mp 121.5–123.2°C; IR (film) *ν*_max_: 3045, 2921, 2872, 1646, 1428, 1098, 1015 cm^–1^; ^1^H NMR (400 MHz, CDCl_3_) δ 1.76–1.89 (m, 4 H), 2.58 (t, *J* = 8.0 Hz, 2 H), 3.02 (t, *J* = 8.0 Hz, 2 H), 3,26 (t, *J* = 6.8 Hz, 2 H), 3.46 (t, *J* = 6.8 Hz, 2 H), 7.30 (d, J = 7.8 Hz, 1 H), 7.28–7.33 (overlap, 1 H), 7.40 (dd, J = 7.8, 7.8 Hz, 2 H), 7.50 (dd, J = 8.0 Hz, 2 H, 7.55 (dd, J = 8.0 Hz, 2 H) ppm; ^13^C NMR (100 MHz, CDCl_3_) δ 24.2, 25.9, 30.6, 36.5, 45.5, 46.4, 126.8, 126.9, 127.0, 128.6, 128.7, 138.8, 140.5, 140.8, 170.5 ppm; MS (ESI, *m/z*): 280 (M + H^+^); HRMS (ESI) calcd for [C_19_H_22_NO]^+^ (M + H^+^): 280.1696; found: 280.1720.

**1–(4–Benzyloxy)benzyl–carbonyl pyrrolidine (17)**

Following the general **method B** (eluent: EtOAc/PE 1:2), amide **17** was obtained (68 mg, 77%) as white crystals: Mp: 115.5–116.8°C; IR (film) *ν*_max_: 3011, 2927, 2863, 1639, 1380, 1089, 1021 cm^–1^; ^1^H NMR (400 MHz, CDCl_3_) δ 1.79–1.94 (m, 4 H), 3.41 (t, *J* = 6.8 Hz, 2 H), 3.48 (t, *J* = 6.8 Hz, 2 H), 3,58 (s, 2 H), 5.04 (s, 2 H), 6.92 (dd, J = 2.2, 6.6 Hz, 2 H), 7.19 (dd, J = 2.2, 6.6 Hz, 2 H), 7.29–7.44 (m, 5 H) ppm; ^13^C NMR (100 MHz, CDCl_3_) δ 24.3, 26.1, 41.3, 45.8, 46.8, 70.0, 114.9, 127.2, 127.4, 127.9, 128.5, 130.0, 137.0, 157.6, 169.7 ppm; MS (ESI, *m/z*): 296 (M + H^+^); Anal. calcd for C_19_H_21_NO_2_: C, 77.26; H, 7.17; N, 4.74. Found: C, 77.41; H, 7.16; N, 4.75.

**1–(2–(4–Benzyloxy)phenyl)ethyl–carbonyl pyrrolidine (18)**

Following the general **method B**(eluent: EtOAc/PE 1:3), amide **18** was obtained (78 mg, 84%) as white crystals: Mp: 78.4–78.8°C; IR (film) *ν*_max_: 3007, 2972, 2943, 2873, 1644, 1508, 1431, 1233, 1018 cm^–1^; ^1^H NMR (400 MHz, CDCl_3_) δ 1.76–1.89 (m, 4 H), 2.51 (t, *J* = 7.2 Hz, 2 H), 2.92 (t, *J* = 7.2 Hz, 2 H), 3,26 (t, *J* = 6.8 Hz, 2 H), 3.44 (t, *J* = 6.8 Hz, 2 H), 5.02 (s, 2 H), 6.89 (d, *J* = 6.6 Hz, 2 H), 7.13 (d, *J* = 8.4 Hz, 2 H), 7.29–7.42 (m, 5 H) ppm; ^13^C NMR (100 MHz, CDCl_3_) δ 24.3, 25.9, 30.2, 36.9, 45.5, 46.4, 69.9, 114.7, 127.3, 127.8, 128.4, 129.3, 133.8, 137.0, 157.0, 170.7 ppm; MS (ESI, *m/z*): 310 (M + H^+^); Anal. calcd for C_20_H_23_NO_2_: C, 77.64; H, 7.49; N, 4.53. Found: C, 77.81; H, 7.50; N, 4.54.

**1–(2–Naphthalenyl)carbonyl pyrrolidine (19) ^(6)^**

Following the general **method A**(eluent: EtOAc/PE 1:2), the amidation of 2–naphthoic acid with pyrrolidine afforded **19** (57 mg, 85%) as white crystals: Mp 57.4–58.5°C; IR (film) *ν*_max_: 3015, 2972, 2879, 1614, 1412, 1018 cm^–1^; ^1^H NMR (400 MHz, CDCl_3_) δ 1.74–1.95 (m, 2 H), 1.95–2.09 (m, 2 H), 3,19 (t, *J* = 6.8 Hz, 2 H), 3,80 (t, *J* = 6.8 Hz, 2 H), 7.47–7.53 (m, 4 H), 7.59–7.99 (m, 3 H) ppm; ^13^C NMR (100 MHz, CDCl_3_) δ 24.3, 26.3, 46.1, 48.5, 124.2, 125.4, 125.5, 126.8, 127.6, 127.9, 129.3, 129.4, 133.6, 136.4, 169.9 ppm; MS (ESI, *m/z*): 226 (M + H^+^).

**1–(2–Naphthalenyl)acetyl pyrrolidine (20)**

Following the general **method A**(eluent: EtOAc/PE 1:5), the amidation of 2–naphthalenyl acetic acid with pyrrolidine afforded **20** (65 mg, 91%) as white wax: IR (film) *ν*_max_: 3020, 2921, 1633, 1421, 1378, 1087, 1021 cm^–1^; ^1^H NMR (400 MHz, CDCl_3_) δ 1.80–1.94 (m, 4 H), 3.45 (t, *J* = 6.8 Hz, 2 H), 3.52 (t, *J* = 6.8 Hz, 2 H), 3.82 (s, 2 H), 7.42–7.48 (m, 3 H), 7.71 (s, 1 H), 7.78–7.82 (m, 3 H) ppm; ^13^C NMR (100 MHz, CDCl_3_) δ 24.4, 26.2, 42.6, 46.0, 46.9, 125.6, 126.1, 127.3, 127.4, 127.61, 127.64, 128.2, 132.4, 132.5, 133.5, 169.5 ppm; MS (ESI, *m/z*): 240 (M+H^+^); HRMS (ESI) calcd for [C_16_H_18_NO]^+^ (M + H^+^): 240.1383; found: 240.1397.

***N*–(Biphenyl–4–ylmethyl)pyrrolidinyl–1–carboxamide (21)**

To a stirred and ice–cold solution of the 4–Biphenylacetic acid (0.5 mmol) in CH_2_Cl_2_ (5 mL), DMF (0.01 mL) and (COCl)_2_ (0.6 mmol, 0.05 mL) were added. After being stirred at 0°C for 1 h, the mixture was concentrated under reduced pressure, keeping the temperature below 30°C. The residue was diluted with dry acetone (5 mL), and added dropwise to a stirred and ice–colded solution of NaN_3_ (1.0 mmoL, 33 mg) in H_2_O (1 mL). The resulting mixture was stirred at 0°C for 30 min, diluted with CH_2_Cl_2_, and washed with brine. The combined organic layers were dried over Na_2_SO_4_, and concentrated under reduced pressure, keeping the temperature below 30°C. The residue was diluted with toluene (15 mL), and reflux for 2 h. The mixture solution was cooled to room temperature, and added dropwise a solution of pyrrolidine (0.06 mL, 0.5 mmol) in toluene (0.5 mL). After being stirred at 80°C for 5 h. the mixture was cooled and concentrated. The residue was purified by flash chromatography on silica gel (eluent: EtOAc/PE 1: 1) to afford compounds **21** (54 mg, 38%) as white solid: Mp 185.2–187.3°C. IR (film) *ν*_max_: 3029, 2924, 2866, 1620, 1524, 1384, 1031, 762, 694 cm^–1^; ^1^H NMR (400 MHz, CDCl_3_) δ 1.84–1.88 (m, 4 H), 3.33 (m, 4 H), 4.45 (d, *J* = 5.6 Hz, 2 H), 4.47 (br, 1 H), 7.32 (dd, *J* = 7.2, 7.2 Hz, 1 H), 7.40 (overlap, 4 H), 7.54 (overlap, 4 H) ppm; ^13^C NMR (100 MHz, CDCl_3_) δ 25.4, 44.1, 45.4, 126.9, 127.06, 127.08, 128.0, 128.6, 139.0, 139.9, 140.7, 156.6 ppm; MS (ESI, *m/z*): 281 (M + H^+^); Anal. calcd for C_18_H_20_N_2_O: C, 77.11; H, 7.19; N, 9.99; Found: C, 77.31; H, 7.18; N, 10.01.

**Biphenyl–4–ylmethyl pyrrolidinyl–1–carboxylate (22)**

To a stirred and ice–cold mixture solution of 4–Phenylbenzyl alcohol (55 mg, 0.3 mmol), Et_3_N (0.08 mL, 0.6 mmol) in anhydrous CH_2_Cl_2_ (4 mL), was added dropwise a solution of bis(trichloromethyl) carbonate (98 mg, 0.33 mmol) in CH_2_Cl_2_ (2 mL) under nitrogen atmosphere. After being stirred at 0°C for 1 h and room temperature for 2 hours, to the mixture solution was added dropwise pyrrolidine (0.05 mL, 0.4 mmol). The resulting mixture was stirred at room temperature for 5 hours, quenched with a saturated aqueous solution of NH_4_Cl and extracted with CH_2_Cl_2_ (3 × 10 mL). The combined organic phases were washed with brine, dried over anhydrous Na_2_SO_4_, filtered, and concentrated under reduced pressure. The residue was purified by flash chromatography on silica gel (eluent: EtOAc/PE 1: 5) to afford compounds **22** (64 mg, 76%) as white crystals: Mp 75.8–76.85°C; IR (film) *ν*_max_: 3017, 2972, 2955, 2882, 1694, 1444, 1422, 1358, 1089, 762, 688, 534 cm^–1^; ^1^H NMR (400 MHz, CDCl_3_) δ 1.83–1.89 (m, 4 H), 3.38–3.44 (m, 4 H), 5.18 (s, 2 H), 7.34 (t, *J* = 7.2 Hz, 1 H), 7.44 (overlap, 4 H), 7.58 (d, *J* =7.2 Hz, 4 H) ppm; ^13^C NMR (100 MHz, CDCl_3_) δ 24.9, 25.7, 45.8, 46.2, 66.3, 127.0, 127.2, 127.3, 128.3, 128.7, 136.1, 140.8, 154.9 ppm; MS (ESI, *m/z*): 282 (M + H^+^); Anal. calcd for C_18_H_19_NO_2_: C, 76.84; H, 6.81; N, 4.98; Found: C, 76.67; H, 6.82; N, 4.97.

**4–Phenylbenzyl cyclopentanyl carboxamide (23)**

To a mixture of 4–Phenylbenzyl amine (55 mg, 0.3 mmol), Et_3_N (0.08 mL, 0.6 mmol) in anhydrous CH_2_Cl_2_ (4 mL) was added dropwise a solution of cyclopentanecarbonyl chloride (44 mg, 0.33 mmol) in CH_2_Cl_2_ (1 mL) under nitrogen atmosphere at 0°C. The resulting solution was stirred at 0°C for 1 h and room temperature for 4 hours, quenched with a saturated aqueous solution of NH_4_Cl and extracted with CH_2_Cl_2_ (3 × 10 mL). The combined organic phases were washed with brine, dried over anhydrous Na_2_SO_4_, filtered, and concentrated under reduced pressure. The residue was purified by flash chromatography on silica gel (eluent: EtOAc/PE 1: 5) to afford compounds **23** (74 mg, 88%) as white crystals: Mp: 151.8–154.6°C; IR (film) *ν*_max_: 3305, 3012, 2947, 2863, 1634, 1549, 1379, 1234, 1114, 1021, 756, 736, 688 cm^–1^; ^1^H NMR (400 MHz, CDCl_3_) δ 1.58–1.63 (m, 2 H), 1.71–1.92 (m, 6 H), 2.56 (m, 1 H), 4.47 (s, 1 H), 4.48 (s, 1 H), 5.85 (br, 1 H), 7.35 (overlap, 3 H), 7.43 (dd, *J* = 7.3, 7.3 Hz, 2 H), 7.56 (overlap, 4 H) ppm; ^13^C NMR (100 MHz, CDCl_3_) δ 25.9, 30.4, 43.2, 45.9, 127.0, 127.3, 127.4, 128.2, 128.8, 137.6, 140.4, 140.7, 176.1 ppm; MS (ESI, *m/z*): 280 (M + H^+^); Anal. calcd for C_19_H_21_NO: C, 81.68; H, 7.58; N, 5.01. Found: C, 81.52; H, 7.60; N, 5.00.

**4–Phenylbenzyl cyclopentanyl carboxylate (24)**

To a mixture of 4–Phenylbenzyl alcohol (55 mg, 0.3 mmol), Et_3_N (0.08 mL, 0.6 mmol) in anhydrous CH_2_Cl_2_ (4 mL), was added dropwise a solution of cyclopentanecarbonyl chloride (44 mg, 0.33 mmol) in CH_2_Cl_2_ (1 mL) under nitrogen atmosphere at 0°C. The resulting solution was stirred at 0°C for 1 h and room temperature for 4 h, quenched with a saturated aqueous solution of NH_4_Cl and extracted with CH_2_Cl_2_ (3 × 10 mL). The combined organic phases were washed with brine, dried over anhydrous Na_2_SO_4_, filtered, and concentrated under reduced pressure. The residue was purified by flash chromatography on silica gel (eluent: EtOAc/PE 1: 30) to afford compounds **24** (77 mg, 92%) as colorless oil: IR (film) *ν*_max_: 3021, 2959, 2863, 1729, 1486, 1377, 1153, 1009, 759, 692 cm^–1^; ^1^H NMR (400 MHz, CDCl_3_) δ 1.55–1.59 (m, 2 H), 1.68–1.73 (m, 2 H), 1.81–1.92 (m, 4 H), 2.79 (m, 1 H), 5.14 (s, 2 H), 7.33 (m, 1 H), 7.42 (m, 4 H) 7.58 (m, 4 H) ppm; ^13^C NMR (100 MHz, CDCl_3_) δ 25.8, 30.0, 43.8, 65.7, 127.0, 127.2, 127.3, 128.4, 128.7, 135.3, 140.6, 141.0, 176.5 ppm; MS (ESI, *m/z*): 281 (M + H^+^); Anal. calcd for C_19_H_20_O_2_: C, 81.40; H, 7.19. Found: C, 81.09; H, 7.18.

**1–(3–Biphenyl–4–ylpropyl)pyrrolidine (25)**

To a stirring suspension of LiAlH_4_ (15 mg, 0.4 mmol) in anhydrous THF (5 mL), was added **16** (84 mg, 0.3 mmol) in anhydrous THF (1 mL) at 0°C. The white suspension was vigorously stirred 1 h at 0°C and 2 h at room temperature, the mixture was cooled to 0°C, and quenched by successive dropwise addition of H_2_O (0.1 mL), 10% NaOH solution (0.1 mL) and H_2_O (0.5 mL). After diluting with methanol (20 mL) the suspension was filtered through celite and washed with methanol for several times. The combined organic layers were dried over anhydrous MgSO_4_, filtered and concentrated under vacuum. The residue was purified by flash chromatography on silica gel (eluent: MeOH/CH_2_Cl_2_ 1: 5) to give compounds **25** (53 mg, 67%) as colorless oil: IR (film) *ν*_max_: 3015, 2920, 1422, 1383, 1112, 1020, 764, 696 cm^–1^; ^1^H NMR (400 MHz, CD_3_OD) δ 1.97–2.00 (m, 4 H), 2.03–2.09 (m, 2 H), 2.73 (t, *J* = 7.6 Hz, 2 H) , 3.02 (t, *J* = 8.1 Hz, 2 H), 3.15 (m, 4 H), 7.32 (overlap, 3 H), 7.43 (overlap, 3 H), 7.55–7.61 (m, 4 H) ppm; ^13^C NMR (100 MHz, CD_3_OD) δ 22.7, 22.8, 27.7, 32.1, 53.3, 54.4, 126.4, 126.7, 126.8, 128.5, 128.6, 139.1, 139.8, 140.8 ppm; MS (ESI, *m/z*): 266 (M+H^+^).

**Supplementary References**

1. Caroline Da Ros Montes D’Oca, Tatiane Coelho, Tamara Germani Marinho, Carolina Rosa Lopes Hack, Rodrigo da Costa Duarte, Pedro Almeida da Silva and Marcelo Gonçalves Montes D’Oca. Synthesis and antituberculosis activity of new fatty acid amides. *Bioorg. Med. Chem. Lett*. **2010**, *20*, 5225–5257.

2. Zheng–Wang Chen, Huan–Feng Jiang, Xiao–Yan Pan, Zai–Jun He. Practical synthesis of amides from alkynyl bromides, amines, and water. *Tetrahedron*. **2011**, *67*, 5920–5927.

3. Yukako Saito, Hidekazu Ouchi, Hiroki Takahata. Carboxamidation of carboxylic acids with 1–tert–butoxy–2–tert–butoxycarbonyl–1,2–dihydroisoquinoline (BBDI) without bases. *Tetrahedron*. **2008**, *64*, 11129–11135.

4. **Masahiko Yamaguchi****, Keisuke Shibato, Hisataka Nakashima, Toru Minami.** Synthesis of phenols by the intramolecular condensation of β, β', δ, δ'–tetraoxoalkanedioates a novel BF_3_–promoted claisen condensation of. *Tetrahedron*. **1988**, *44*, 4767–4775.

5. Matthew H. Todd, Steven F. Oliver, and Chris Abell. A Novel Safety–Catch Linker for the Solid–Phase Synthesis of Amides and Esters. *Org.Lett*.**1999**, *1*, 1149–1151.

6. Kekeli Ekoue–Kovi and Christian Wolf. Metal–Free One–Pot Oxidative Amination of Aldehydes to Amides. *Org. Lett*. **2007**, *9*, 3429–3432.
